# Improved Performance of Catalysts Containing Pt, Pt–Sn, and V in the Dehydrogenation of *n*-Butane by Radio-Frequency Induction Heating

**DOI:** 10.1021/acssuschemeng.4c10045

**Published:** 2025-02-11

**Authors:** Cameron L. Roman, Jonathan Lucas, James A. Dorman, Kerry M. Dooley

**Affiliations:** † Department of Chemical Engineering, Louisiana State University, Baton Rouge, Louisiana 70803, United States

**Keywords:** dehydrogenation, *n*-butane, radio-frequency induction heating, core−shell catalysts

## Abstract

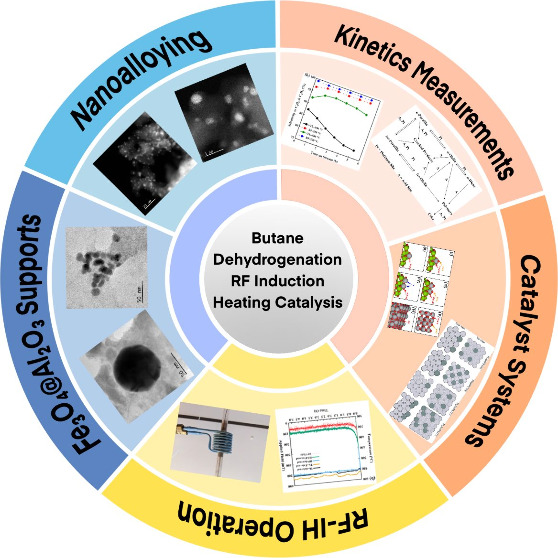

Due to an abundance of shale gas, manufacturers are interested in meeting increased demands for alkenes, especially *n*-alkenes, by the dehydrogenation of light alkanes. While the catalysts for these reactions have been studied for many years, alkene selectivity and deactivation remain challenging problems. This work addresses these problems by the substitution of a localized indirect heating method (radio-frequency induction heating, RF-IH) for more traditional process heating employing steam or burners (e.g., natural gas combustion). RF-IH has been applied to the dehydrogenation of *n*-butane to C_4_ alkenes by utilizing magnetically susceptible catalysts based on Fe/Fe_3_O_4_ susceptors. Magnetic core–shell catalysts with either Pt or V as active metals were synthesized to mimic typical *n*-butane dehydrogenation catalysts. For these catalysts, RF-IH operation resulted in significantly improved selectivity to alkenes and less deactivation when compared to conventional thermal heating, although the initial activities were not always as high as their thermally operated counterparts. These results provide motivation to continue investigating the effects of RF-IH and its benefits to certain heterogeneous catalytic processes.

## Abstract 2

Due to an abundance of shale gas, manufacturers are interested in meeting increased demands for alkenes, especially *n*-alkenes, by the dehydrogenation of light alkanes. While the catalysts for these reactions have been studied for many years, alkene selectivity and deactivation remain challenging problems. This work addresses these problems by the substitution of a localized indirect heating method (radio-frequency induction heating, RF-IH) for more traditional process heating employing steam or burners (e.g., natural gas combustion). RF-IH has been applied to the dehydrogenation of *n*-butane to C_4_ alkenes by utilizing magnetically susceptible catalysts based on Fe/Fe_3_O_4_ susceptors.

## Introduction

### Catalytic Dehydrogenation, with and without Radio-Frequency Induction Heating

Catalytic dehydrogenation of alkanes provides a route from low-cost saturated hydrocarbon feedstocks to olefins.^1^ The demand for polymers, oxygenates, and other chemical intermediates is only expected to grow, increasing the demand for olefins made from light alkanes.^2−4^ However, catalyst selectivity and deactivation remain challenging problems in alkane dehydrogenations. Transition-metal catalysts typically employ Pt or Cr as their active metal, and the Pt usually alloyed or mixed with another transition metal or semimetal (sometimes called a “promoter”). Cheaper alternatives to Pt are being explored, with V being a possible candidate, but its quick deactivation makes it challenging to implement commercially. It is thought that Cr-based catalysts will eventually be phased out due to Cr’s toxicity in water.

The dehydrogenations are highly endothermic and often both thermodynamically and kinetically limited.^1,2,5^ High temperatures and low pressures are required for acceptable conversion (30–50%).^1,2,6^ The surface intermediates are highly reactive and can undergo deep dehydrogenation, isomerization, cracking, and coking.^2,3,7^ A typical commercial catalyst for propane or butane dehydrogenation is a mixture or alloy of Pt–Sn, where the Sn promoter increases selectivity.^3^ Promotion by Sn has been heavily studied, with it being found to increase the activity, selectivity, and catalyst life. The increase in performance is attributed to one or both geometric and electronic affects.^3,5,8^ From a geometric perspective, Sn can cover the low coordination sites [Pt(211) and Pt(100)] that promote coke production and disrupt the large Pt–Pt ensembles that allow production of graphene on Pt(111).^3,9−13^ Evidence comparing catalysts before and after butane dehydrogenation suggests that Pt alloys sinter at a slower rate than Pt.^3,9,10^ From an electronic perspective, it is commonly accepted that alloyed Sn species transfer electrons to the Pt 5d band.^2,3,6,8^ Density functional theory (DFT) calculations confirm that Sn and similar promoters can weaken the binding energies of both product alkenes and coke precursors.^2,3,5,14,15^

The drive to couple renewable electrical production to process heating has led to the substitution of localized heating for traditional process heating employing steam or burners (e.g., natural gas combustion). One promising localized method is radio-frequency induction heating (RF-IH), based on alternating magnetic fields that provide the energy for magnetically susceptible catalysts or catalyst components to heat locally.^16^ This approach has been adapted to many common reactions such as biomass pyrolysis,^17^ reforming of methane,^18^ and propane dehydrogenation,^19,20^ all of which have displayed some evidence of activity and selectivity improvements. Other potential applications have been discussed in a review.^21^

RF-IH could alleviate two problems facing light alkane nonoxidative dehydrogenation. Significant efforts have been made to prevent the reactor temperature from deviating more than 50–60 °C from the target. This normally requires heating the feed above 550 °C.^22^ Due to the direct and almost instantaneous heating of RF-IH, it could not only reduce the difference between the target and feed temperature but also eliminate the need for such extensive preheating. Because coking continues to be one of the main issues facing dehydrogenation catalysts, RF-IH could reduce or even possibly eliminate coking and extend the catalyst lifetime.

Marbaix et al.^20^ synthesized a Pt–Sn propane dehydrogenation catalyst utilizing a FeCo alloy as the susceptor at 525–705 °C, with a Pt–Sn catalyst supported on SiO_2_–Al_2_O_3_.^20^ The dehydrogenation [*P*_propane_ = 1 bar; weight hourly space velocity (WHSV) = 5.7 h^–1^ at STP, 300 kHz] gave conversions from 9.1 to 17.5% with a C_3_H_6_ selectivity from 43.3 to 80.3% during 1 h at 40 mT field strength. They reported a steady wall temperature of 350 °C. To increase the conversion, the magnetic field was increased to 60 mT, but the temperature quickly surpassed 800 °C with no measurable propylene production. TEM post-mortem analysis of the catalyst revealed extensive sintering of the FeCo nanoparticles (NPs). This led them to investigate the FeCo sintering at 40 mT. At 40 mT, the mean particle diameter grew from 10.6 to 15.5 nm [1.5 h time-on-stream (TOS)] to 53.6 nm (3.5 h TOS), with coke deposition and Pt–Sn sintering. These observations revealed two significant and related issues that must be addressed in RF-IH dehydrogenation: reducing susceptor sintering and better temperature control.

Martínez-Prieto et al.^19^ explored propane dehydrogenation via RF-IH by encapsulating the FeCo (5 wt %) and Co (5 wt %) NPs in a carbon support impregnated with Pt–Sn.^19,20^ The results for *P*_propane_ = 1 bar and WHSV = 1.9 h^–1^ at STP were^19^ 2.7% initial conversion, 88.1% propylene selectivity, and 530 °C measured bulk temperature.^19,23^ Increasing the field strength to 65 mT gave 620 °C bulk temperature, 10.4% conversion, and 79% selectivity. These conversions are well below calculated equilibrium conversions.^19,23^ The local surface temperature was likely to be higher than the measured bulk temperature. It has been reported that the surface of the magnetic NPs can be ∼60–70 °C hotter than an insulating oxide support.^24,25^ The encapsulation of FeCo and Co did increase the overall catalyst stability, but sintering of Pt–Sn was observed. In summary, the susceptor’s encapsulation increased catalyst stability, but conversion and selectivity were less than those of traditional dehydrogenation catalysts at comparable temperatures.^3^

Comparing this RF-IH dehydrogenation catalyst to a comparable conventionally heated one, Phan et al.^26^ studied Pt–Sn/γ-Al_2_O_3_ for propane dehydrogenation (WHSV = 3.2 h^–1^; *P*_propane_ = 1 bar, 600 °C). At 6 h TOS, the conversion was 37%, with 94% selectivity. After 10 reaction cycles, the conversion was 14%, and the selectivity was 83%.

### Magnetic NPs for RF-IH

RF-IH directly transfers energy to a target metallic or magnetic material, a susceptor, to heat the material locally. Because there is effectively no heat transfer through reactor walls and reactants, the catalyst core can reach target temperatures almost instantly. Abu-Ladan et al.^27^ studied the hydrodeoxygenation of biomass bio-oil vapor, comparing RF-IH to conventional heating. They observed deoxygenation with a decreasing oxygen–carbon ratio, but with conventional heating, essentially no deoxygenation was noted, and there was rapid coking. Other researchers deployed nanoparticulate NiCo catalysts^28−30^ for highly endothermic steam reforming and dry reforming of methane,^28^ achieving both high methane conversions (>98%) and high temperatures (∼780–800 °C) in steam reforming. A similar approach has been attempted with microwave heating in dry (CO_2_) reforming, where a reduction of coke formation has been observed.^31^

RF-IH is characterized by multiple mechanisms to convert the absorbed energy from the alternating magnetic field to heat, the most important of which is hysteresis heating. Within a specific diameter range, atomic magnetic moments (single domain) or domain walls (multiple domains) rotate under an alternating magnetic field.^32−37^ These domains are characterized by specific anisotropy energies, and when the energy is high relative to the frequency and amplitude of the field applied, the rotation is inhibited, resulting in hysteresis.^32^ The rotation is accompanied by energy losses released as heat. Additionally, these materials possess a critical diameter (*D*_c_) below which the single domain exists while multidomains form as the NP size grows.^34,37,38^ This critical diameter provides the highest coercivity (resistance of the material to changes in magnetization).^36,37^

**Figure 1:**
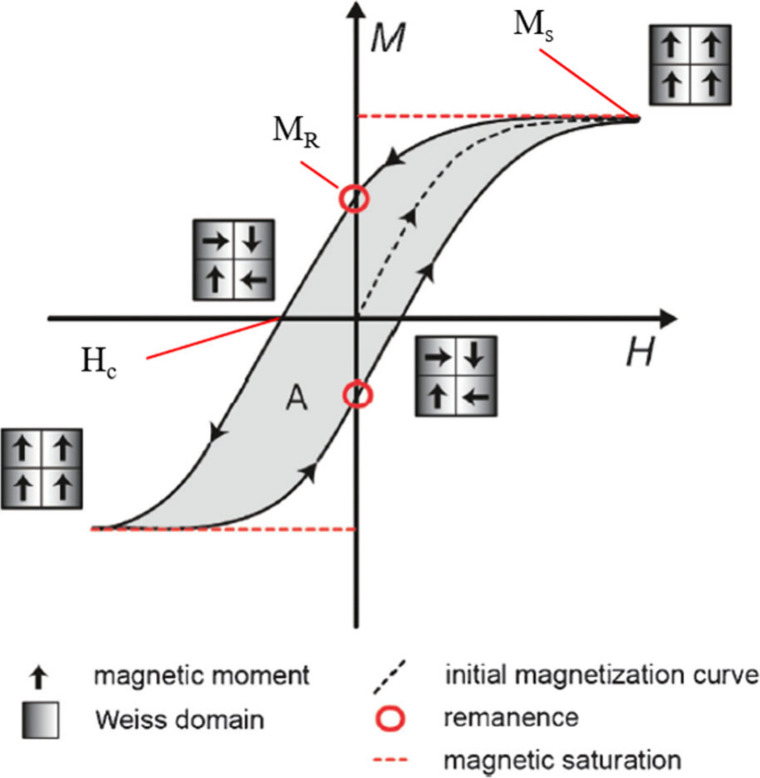
Hysteresis loop of single-domain or multidomain ferromagnetic or ferrimagnetic material.^33^*H* is the applied field and *M* the magnetization. Reproduced from with permission from ref (33). Copyright 2012 Oxford University Press.

Heating relies on the area of and the oscillation rate of the NPs’ hysteresis loop. In an alternating magnetic field, the magnetic (Weiss) domains align themselves in the direction of the applied field (Figure 1).^32,33,39^ Once the field is removed, a magnetization level remains, *M*_R_, the remanent magnetization.^32,33,39^ The opposing field to overcome this resistance and bring it to net-zero magnetization is the coercivity, *H*_c_.^32,33,39^ As the material returns to zero magnetization, the magnetic moments return to a lower energy level, releasing heat (eq 1).^32,33,39^
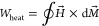
1*W*_heat_ represents the system’s net work or the quantity of heat produced.^32^ Anisotropy increases coercivity and, therefore, the total possible heat.^32^ The magnitude of the applied field must increase to overcome the anisotropy energy.^32^ If the field can no longer overcome this energy barrier, then reversal of the magnetic moment is impossible and no heat will be generated.

A popular susceptor and catalyst support in RF-IH is ferrimagnetic Fe_3_O_4_ (4.0 μ_B_), possessing a relativity high anisotropy energy, low cost, low toxicity, accessible *D*_c_ (128 nm, although particles as small as 20 nm have shown hysteresis losses), and a high Curie temperature (*T*_c_ = 557–583 °C).^34,35,38,40−43^ A high *T*_c_ value is required for two reasons. As the temperature increases, *M*_s_ and therefore the potential heat generation decrease.^42^ Once the material reaches *T*_c_, thermal energy overcomes the exchange interaction between the magnetic moments, and the coercivity decreases to zero, with the material becoming paramagnetic.^32,37^ At temperatures near its *T*_c_, Fe_3_O_4_ can be easily reduced to metallic Fe, whose *T*_c_ is much higher, 770 °C, and whose *D*_c_ is much lower, 15 nm.^34,38^

RF-heated catalysts can be 90% energy efficient in an industrial environment compared to 50% for a conventional reactor.^44^ The increased temperature uniformity can prevent hot-spot generation, which can lead to unwanted side reactions.^32,33,37^ However, the constraints imposed by the magnetic material increase the difficulty of designing an effective catalyst. The active metal or metal oxide crystallite size and morphology can influence hysteresis losses, and their control in optimal catalyst design can conflict with the demands of induction heating.^28,45^ Missing are rigorous comparisons using the same catalysts of RF-IH to conventional thermal heating for complex catalytic processes, those that can provide a real measure of the capabilities of RF-IH to affect catalyst activity, selectivity, and stability. This need is addressed here by exploring the dehydrogenation of *n*-butane to alkenes using magnetically susceptible catalysts based on Fe/Fe_3_O_4_ susceptors.

The equilibrium conversion of *n*-butane to 1-butene is about 45% at 550 °C (1.01 bar, ASPEN-HYSYS). Five dehydrogenated products are possible: 1-butene, *cis*-2-butene, *trans*-2-butene, isobutene, and 1,3-butadiene.^46−51^ 1-Butene is typically more valuable than 2-butene because some applications require high-purity 1-butene.^50^ The diene finds most of its use in the production of styrene–butadiene rubber.^50^ Frey and Huppke^46^ analyzed the equilibria of the butene isomers formed using a chromium oxide catalyst. Dehydrogenation formed three of the four alkenes (no isobutene), with *trans*-2-butene being the most abundant, in accordance with tabulated free energies. Temperature changes have a minimal impact on the product distribution. Different types of catalysts are needed to produce more of the diene.^52^

## Methods

### Materials

The preparation details for the magnetic NP cores with carbon and Al_2_O_3_ shells are given in the Supporting Information.

Fe_3_O_4_@C@γ-Al_2_O_3_/Pt (0.5 wt %), denoted as FCA/Pt, was prepared by incipient wetness impregnation of FCA-1 with aqueous [Pt(NH_3_)_4_](NO_3_)_2_. It was dried at 100 °C for 12 h and then at 550 °C (10 °C min^–1^) for 4 h in N_2_ and then at 550 °C for 2 h in 5% H_2_. Fe_3_O_4_@C@γ-Al_2_O_3_ /Pt (0.5 wt %)-Sn (1.0 wt %) (FCA/Pt-Sn) was prepared by incipient wetness sequential coimpregnation (IWC) of FCA-2, first with tetrabutyltin/pentane under N_2_, and drying under N_2_ for 12 h at 100 °C. Then aqueous Pt(NH_3_)_4_](NO_3_)_2_ was added in a similar manner. The same high-temperature treatments as those for FCA/Pt were used.

IWC was also used for Fe_3_O_4_@C@γ-Al_2_O_3_/VO_*x*_ (3.0 wt % V) (FCA/VO_*x*_). FCA-3 was impregnated with a solution of NH_4_VO_3_ in equimolar oxalic acid, dried at 90 °C for 12 h, and then dried at 550 °C (10 °C min^–1^) for 2 h in N_2_ and 550 °C for 2 h in 5% H_2_.

### Structural Characterization

The amount of carbon encapsulation was characterized by thermogravimetry/differential scanning calorimetry (PerkinElmer STA 6000) using temperature-programmed oxidation (TPO). The sample was equilibrated at 100 °C under N_2_, switched to air, and ramped at 2.0 °C min^–1^ to 700 °C. Powder X-ray diffraction (XRD) data were obtained using Cu Kα_1_ (λ = 1.542 Å) emission on a PANalytical X-ray diffractometer with a step size of 0.026° and a dwell time of 0.72 s for the fresh catalyst and Cu Kα_1_ emission also on a Rigaku X-ray diffractometer with a step size of 0.025° and a dwell time of 1.0 s for the spent catalyst.

Surface area and pore volume measurements were performed on a Micrometrics ASAP 2020 instrument utilizing the Brunauer–Emmett–Teller (BET) method. The carbon and hydrogen contents postreaction were quantified by Galbraith Laboratories (Knoxville, TN) using combustion analysis. Samples for inductively coupled plasma optical emission spectrometry (ICP-OES; PerkinElmer Optima 8000) analyses were prepared by digesting the fresh catalysts in aqua regia under mild heating. Pt dispersion was quantified by 25 °C pulse chemisorption on an Anton Paar ChemBET Pulsar with H_2_ (assumed 1 H/Pt).

X-ray absorption spectra (both XANES and XAFS) were taken at the LSU Center for Advanced Microstructures and Devices (CAMD) WDCM beamline with a Si(111) crystal monochromator. The integration time was adjusted to obtain adequate counts up to *k* = 12 Å^–1^. The Pt L_III_-edge spectra were collected at room temperature in fluorescence mode with a Pt foil (*E*_0_ = 11564 eV) calibration standard. Data were processed (background subtraction, deglitching, and merging of three spectra) using *Athena 0.8.061*.^53^ Pt L_III_-edge XAFS fitting was performed in *Artemis*, version 0.9.26. Three parameters were varied to obtain best fits of the data to various bulk structures: σ^2^ (the Debye–Waller factor), Δ*E*_0_ (deviation in *E*_0_ caused by structural deviations from the ideal crystal structure), and Δ*R* (deviation in the interatomic distance). A Pt foil standard was fit first to obtain *S*_0_^2^ (amplitude reduction factor). The fitting range in *R* space was 1–5 Å, and all significant scattering paths, as identified by *Artemis*, were included.

Catalyst morphology was imaged by scanning electron microscopy (SEM) and transmission electron microscopy (TEM). SEM was performed using a Quanta 3D field-emission gun focused ion beam (FEG FIB)/SEM dual-beam system with acceleration voltages between 500 and 30 kV. Samples were Pt-sputtered. Initial TEM was performed using a JEOL JEM-1400 operating at 120 kV and an Orius Camera SC1000A 1 with a 0.20 nm lattice image and 0.38 nm point image resolution. The samples were dispersed in toluene and drop-cast onto a 300-mesh lacey carbon grid. High-resolution TEM (HRTEM) was performed at Oak Ridge National Laboratory using a 200 kV JEOL NEARM microscope with double aberration correctors, a dual-energy-loss spectrometer, and a cold FEG source. Samples were dispersed in ethanol and drop-cast onto a 300-mesh lacey carbon grid.

X-ray photoelectron spectroscopy (XPS) spectra were collected using a Scienta Omicron ESCA 2SR X-ray photoelectron spectrometer equipped with a monochromatic Al Kα (*h*ν = 1486.6 eV) X-ray source and a hemispherical analyzer with a 128-channel detector. The inherent Gaussian width of the photon source was 0.2 eV. The pressure inside the chamber was 1.5 × 10^–9^ Torr. XPS energies were calibrated to the adventitious C 1s peak at 284.8 eV, and peak deconvolution was performed (using the *CasaXPS* software) assuming Gaussian–Lorentzian peaks after Shirley background subtraction.

### Catalytic Performance Evaluation

The RF-IH was provided by an Ambrell EASYHEAT 8130LI 10 kW induction heater (0–600 A, 224 kHz) with an eight-turn, 25-mm-diameter Cu coil. Specific loss power (SLP) measurements were performed on the fresh catalysts, 20 mg of sample per 1 mL of deionized (DI) H_2_O. Samples were loaded into 5 mL glass vials and heated in an applied field of 121 mT. The reaction of *n*-butane was performed in a system with a 125 × 560 mm quartz vertical packed-bed reactor, as shown in Figure S1. Upstream, the tube was packed with 4.8 mm SiC pellets and heated externally to 240 °C. The catalyst bed was packed between four 9.4 × 10.7 mm cordierite monoliths and quartz wool. A 3.0 mm sand-filled thermowell was inserted through the monoliths into the catalyst, containing a ^1^/_32_-in. (0.79 mm) N-type thermocouple. A second N-type thermocouple was centered on the external wall, and another K-type thermocouple was placed near the reactor exit. For RF-IH operation, the temperature controller varied the amperage of the induction heater. An emergency switch was installed to stop runaway heating. The reactor is shown in Figure S2. Further details are shown in the Supporting Information and in a thesis.^54^

For the FCA/Pt and FCA/Pt-Sn runs, 0.75 g of catalyst was used. For FCA/VO_*x*_, 0.75 and 1.0 g were used. After N_2_ purging, the reactor was heated to the reduction temperature. Reduction was with 5% H_2_ (balance N_2_) at 30 mL min^–1^ for 2 h, and then the reactor was purged with N_2_ again.

The reactions were performed at 555 and 505 °C for 5 h. For FCA/Pt and FCA/Pt-Sn, *n*-butane (Airgas, >99% *n*-butane) and H_2_ were fed into the reactor at WHSV = 2.65 h^–1^ and a H_2_/*n*-butane molar ratio of 1.25:1. For FCA/VO_*x*_, the WHSV values were 2.04 h^–1^ (0.75 g of catalyst) and 1.22 h^–1^ (1.00 g of catalyst). The products were analyzed by an Agilent GC 6890 instrument using a Supel Q-PLOT (30 m × 0.32 mm) column joined to a Chrompack PLOT fused-silica CP-Al_2_O_3_/KCl (15 m × 0.32 mm) column. Further details are shown in Figures S3 and S4 and in a thesis.^54^

## Results and Discussion

### Fresh Catalyst Characterization

**Figure 2:**
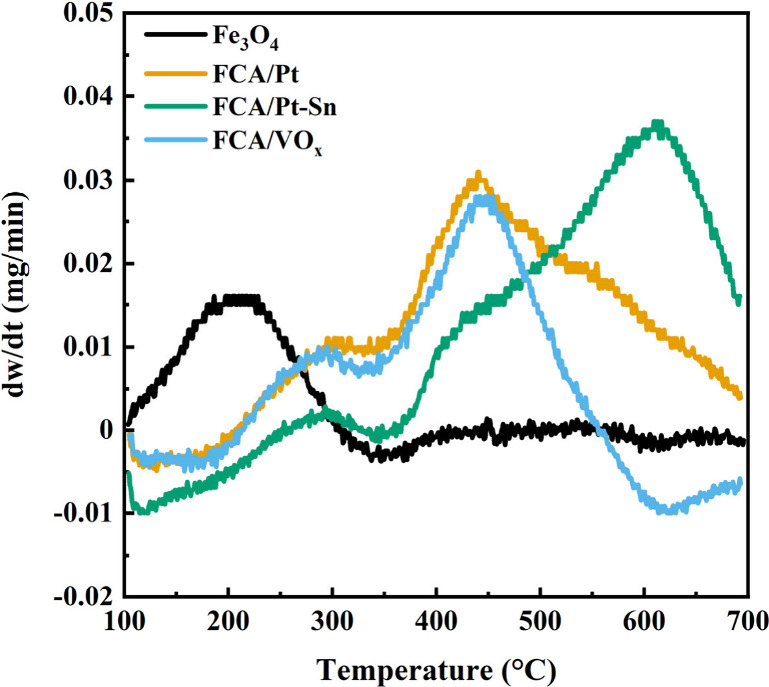
TPO (air, 2 °C/min) weight derivatives for starting commercial Fe_3_O_4_ and the catalysts (FCA/Pt, FCA/Pt-Sn, and FCA/VO_*x*_).

TPO was used to confirm carbon encapsulation of iron oxide (Figure 2). With the starting material, Fe_3_O_4_, the peak weight gain due to oxidation occurred around 200–250 °C. It has been reported that Fe_3_O_4_ is stable in air up to 250 °C.^40^ For the core–shell catalysts (FCA/Pt, FCA/Pt-Sn, and FCA/VO_*x*_), the peak weight gain shifted to above 400 °C. Therefore, TPO confirms that the carbon shell was successfully formed.^55^ Martínez-Prieto et al.^19^ found that encapsulation of magnetic NPs can significantly reduce sintering under reaction conditions and prevent thermal runaway under RF heating.

**Figure 3:**
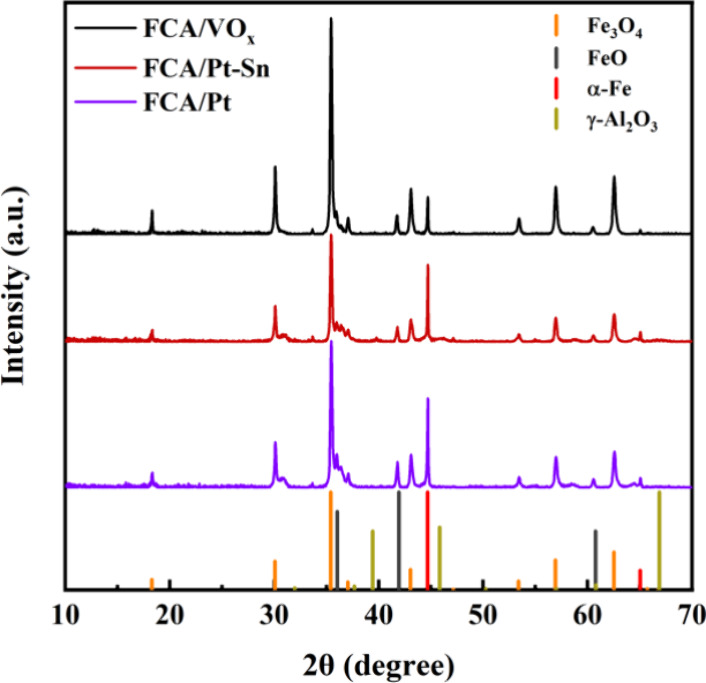
XRD patterns of FCA/Pt, FCA/Pt-Sn, and FCA/VO_*x*_. The drop-line marks the reference patterns.

XRD revealed three iron phases in the reduced core–shell catalysts: Fe_3_O_4_, FeO, and α-Fe (Figure 3). Peaks at 2θ = 18.3, 30.1, 35.5, 37.1, 43.1, 47.0, 53.5, 56.9, 62.5, and 65.1° are characteristic of Fe_3_O_4_ (ICDD: 01-072-2303), while the other peaks are representative of FeO (ICDD: 00-006-0615) and α-Fe (ICDD: 00-006-0615). For γ-Al_2_O_3_ (ICDD: 00-010-0425), major peaks are expected at 39.4, 45.9, and 66.9° but are not present, suggesting that Al_2_O_3_ is amorphous. XRD patterns comparing the starting Fe_3_O_4_ to Fe_3_O_4_@C core–shell materials show a large fraction of Fe_3_O_4_ reducing to FeO and α-Fe during carbonization (Figure S5a). In contrast, the heat treatment that formed Al_2_O_3_ causes oxidation back to Fe_3_O_4_ (Figure S5b), suggesting that heat treatments in the 500–600 °C range, even in N_2_, gradually oxidize the core. Returning to the freshly prepared catalysts, the dominant peak of each phase was used to calculate the average crystallite size using the Debye–Scherrer equation (Table 1). For all three catalysts, the average size for Fe_3_O_4_ is 44–46 nm, and while it is the dominant phase, the relative peak area for the dominant (311) Fe_3_O_4_ peak at 35.4° increased from 64 to 85% upon going from FCA/Pt and FCA/Pt-Sn to FCA/VO_*x*_. The FeO phase is similar for all catalysts, 39–45 nm in size and 10% or less peak area. FCA/Pt and FCA/Pt-Sn contain a similar fraction of α-Fe (26–28%), but FCA/VO_*x*_ contains much less after reduction, suggesting that Pt is promoting additional reduction by H_2_ spillover. The average crystallite size of α-Fe is 78–92 nm.

**Table 1:** Average Crystallite Sizes (nm) and Relative Peak Areas (%) for FCA/Pt, FCA/Pt-Sn, and FCA/VO_*x*_ Using XRD Data

	Fe_3_O_4_ (311)		FeO (200)		α-Fe (110)	
catalyst	avg. crystallite size (nm)	peak area (%)	avg. crystallite size (nm)	peak area (%)	avg. crystallite size (nm)	peak area (%)
FCA/Pt	45	64	45	10	80	26
FCA/Pt-Sn	46	64	43	8	92	28
FCA/VOx	44	85	39	7	78	9

FeO is antiferromagnetic and provides no hysteresis heating.^56^ On the other hand, while α-Fe can be beneficial, because its coercivity is higher than Fe_3_O_4_, the average crystallite size here is 5 times the optimal *D*_c_, suggesting that α-Fe NP fractions are deep into the multidomain phase and would only provide a small fraction of the total coercivity.^34,35,41,57,58^

BET surface area and pore volume measurements were performed on each of the supports and core–shell catalysts (Table 2) with the isotherms shown in Figure S6a. FCA-1, compared to FCA-2 and FCA-3, has roughly 70% of the surface area and 80–70% of the pore volume, suggesting some inconsistency with the Al_2_O_3_ shell synthesis. The pore widths for the support ranged from 2 to 8 nm, as shown in Figure S6b. The absence of larger pores demonstrates the effectiveness of the carbon shells in sealing off the Fe_3_O_4_ cores. The impregnation of Pt and Pt–Sn into the supports decreased somewhat the surface area and pore volume. However, there were no major changes in the distribution of the pore widths.

**Table 2:** Textural Properties of Catalysts and Corresponding Supports, Elemental and Compound Analysis, and H_2_ Dispersion Measurements

support/catalyst	surface area (m^2^/g)	pore volume (cm^3^/g)^a^	C (wt %)	H (wt %)	Al_2_O_3_ (wt %)	dispersion % (H_2_)
FCA-1	72	0.160				
FCA/Pt	48	0.139	<0.50	<0.50	16	20
FCA-2	103	0.193				
FCA/Pt-Sn	79	0.142	1.11	<0.50	14	12
FCA-3	107	0.220				
FCA/VO_*x*_	49	0.121	0.53	<0.50	16	

a Mesopores only, with no significant micropore volume measured.

With the FCA-3 support, a 3.0 wt % V loading should result in a theoretical vanadium coverage of 3.4 V/nm^2^ and a mixture of mono- and polyvanadate structures.^59^ The impregnation halved the surface area and the pore volume. The pore width distribution of FCA/VO_*x*_ showed mostly smaller pores, suggesting that most of the vanadium crystallized in the larger pores.

Combustion analysis on FCA/Pt, FCA/Pt-Sn, and FCA/VO_*x*_ reveals that carbon makes up less than 1.2 wt % of the catalysts (Table 2). The calculated Al_2_O_3_ weight percent values are below the target of 20 wt %, even factoring in the weight of the active metals. There are two possibilities for the shortfall. First, the Al_2_O_3_ encapsulation involves submerging the Fe_3_O_4_@C NPs into a sol, and possibly attachment of the alumina precursor is less than 100% efficient. Second, as mentioned earlier, carbonization caused a partial reduction of the core, but later heat treatment caused some of the reduced core to reoxidize back to Fe_3_O_4_. Because the target amount of the Al_2_O_3_ precursor was measured with respect to the mass of the carbonized material, any later oxidation of the core with an accompanying increase of mass would cause the Al_2_O_3_ weight percent to decrease.

The measured Pt dispersion decreases from 20 to 12% with the addition of Sn (Table 2). Typically, the decrease in H_2_ chemisorption is caused by the alloying of Pt–Sn.^6,60^ Due to the low loading of Pt on FCA/Pt-Sn, which makes XPS difficult, XANES and XAFS Pt L_III_-edge spectra were taken. The measured Pt spectrum of FCA/Pt-Sn is nearly identical to that of Pt foil (white line ∼11570 vs 11569 eV for Pt foil); therefore, Pt is Pt^0^ (Figure S7a). Examining the XAFS spectra (Figure S7b), it appears that a Pt atom is still surrounded mainly by other Pt atoms because the fit for Pt metal is better than the fit to any of the Pt-rich Sn alloys (Pt_3_Sn, PtSn, or Pt_2_Sn_3_). Fits to the Sn-rich alloys were even worse. For example, to minimize the *R* value of a fit to a Pt_3_Sn structure, the Pt coordination number (*N*_1_) was regressed to 12 and the Sn coordination number (*N*_2_) to 2.5, whereas the normal coordination numbers in Pt_3_Sn are 8 and 4. This suggests that the average Pt atom sees far more Pt than Sn atoms immediately adjacent to it. The *N*_1_ value of a fit to Pt metal was only 9.3 vs 12 for large Pt crystals. This means that the Pt domains are small. According to the DFT-based correlations of Beale and Weckhuysen,^61^ a standard half-sphere model of a Pt crystallite is consistent with a 9.3 *N*_1_ value for a crystallite of ∼300 Pt atoms, or a domain size of 2.9 nm.

**Figure 4:**
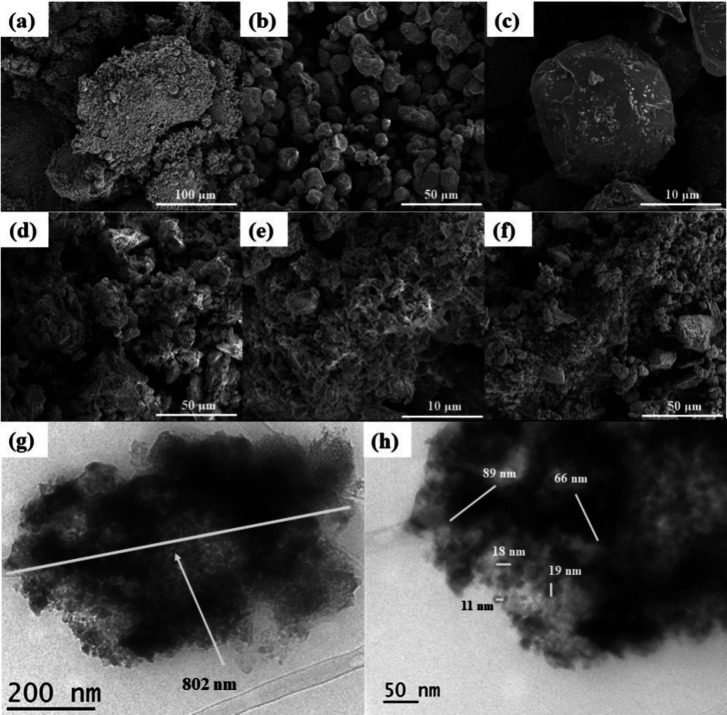
SEM images of as-synthesized materials: (a) Fe_3_O_4_; (b) Fe_3_O_4_@C-1; (c) magnified Fe_3_O_4_@C-1; (d) FCA/Pt; (e) FCA/Pt-Sn; (f) FCA/VO_*x*_. (g and h) TEM images of FCA/Pt–Sn.

The SEM images of the starting Fe_3_O_4_ (Figure 4) reveal what looks like large amorphous particles. The completely dark regions are pores or channels between smaller particles. Agglomeration is more pronounced in magnetic NPs due to their magnetic dipole–dipole attraction and remanent magnetization or residual magnetism.^62,63^ Therefore, it is not unexpected for the smaller Fe_3_O_4_ particles to assemble into larger particles. Adding the carbon shell separates the core–shell particles into smaller aggregates that are polyhedron-shaped (Figure 4b,c). The size distribution appears to be relatively narrow.

**Figure 5:**
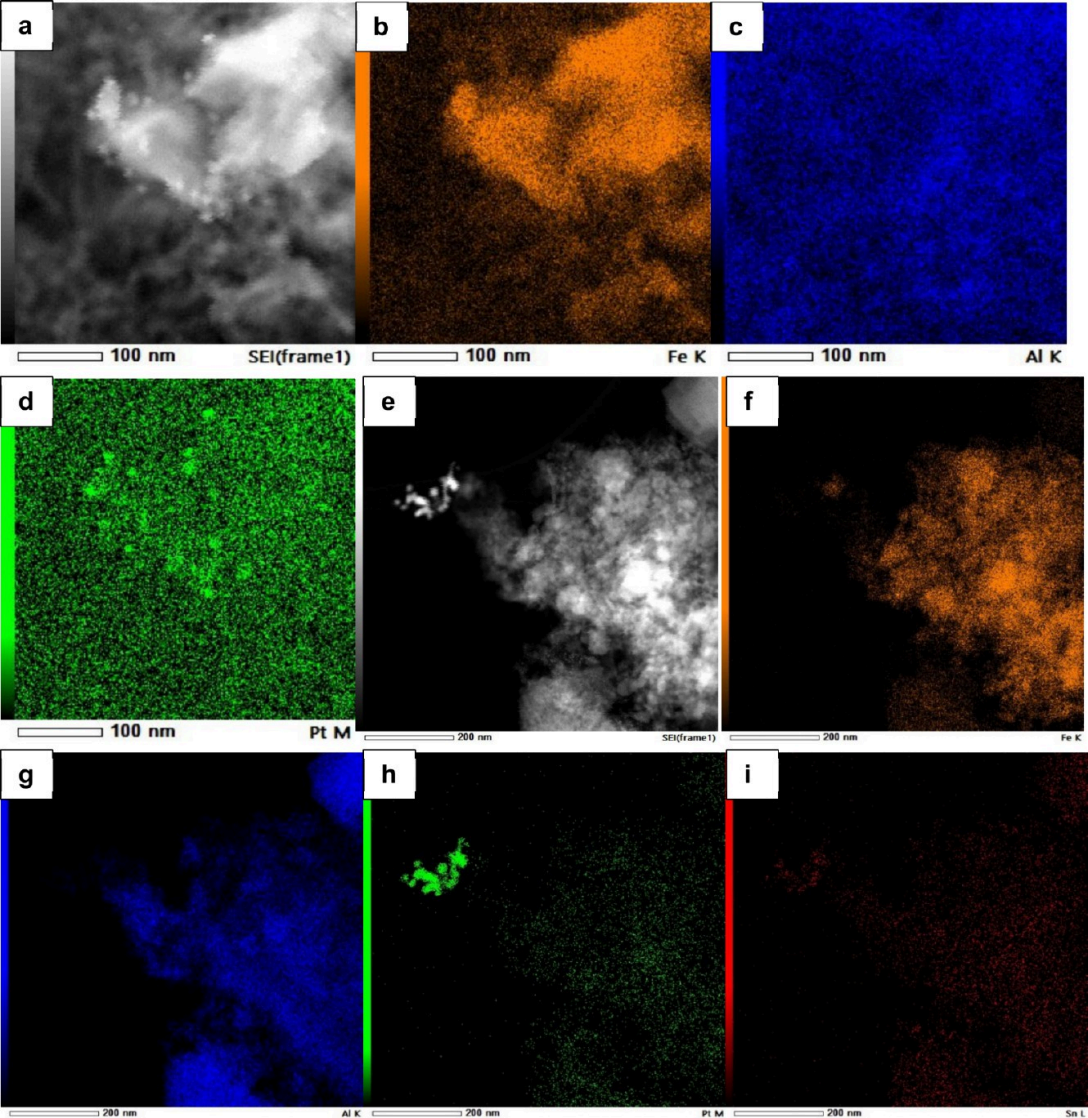
SEM images and EDS maps of fresh FCA/Pt and FCA/Pt-Sn catalysts: (a and e) SEM images; (b and f) Fe EDS; (c and g) Al EDS; (d and h) Pt EDS; (i) Sn EDS.

The catalysts FCA/Pt (Figure 4d), FCA/Pt-Sn (Figure 4e), and FCA/VO_*x*_ (Figure 4f) were also imaged. The addition of Al_2_O_3_ appears to create larger particles from Al_2_O_3_ encapsulating many of the smaller Fe_3_O_4_@C particles together. The highly textured surface is typical of amorphous Al_2_O_3_. Mahendra et al.^64^ and Ammar et al.^65^ observed similar features in their synthesis of Fe_3_O_4_@Al_2_O_3_. The TEM image of FCA/Pt-Sn shows one of these encapsulated particles, with the darker contrast being Fe_3_O_4_@C and the lighter contrast being Al_2_O_3_ (Figure 4g). This aggregate is over 800 nm in width. However, from Figure 4g,h, the size of the Fe_3_O_4_@C cores is mostly in the 10–90 nm range. It appears that the as-synthesized catalyst particles contain a wide distribution of magnetic cores, with most likely various levels of coercivity. However, regardless of this distribution, an irregular core–shell structure of the catalysts is evident, as observed in the SEM images and energy-dispersive spectroscopy (EDS) maps of Figure 5 and in the SEM images of Figure S8, which give some quantification of core variation. Note especially the evidence of many small Pt crystallites in both samples (Figure 5), the overlapping nature of Pt and Sn in FCA/Pt-Sn, the concentrated Fe regions (cores), the far more diffuse Al regions (shells), and the presence of some Fe outside the cores and in the shell regions and also that of some Pt in both regions.

**Figure 6:**
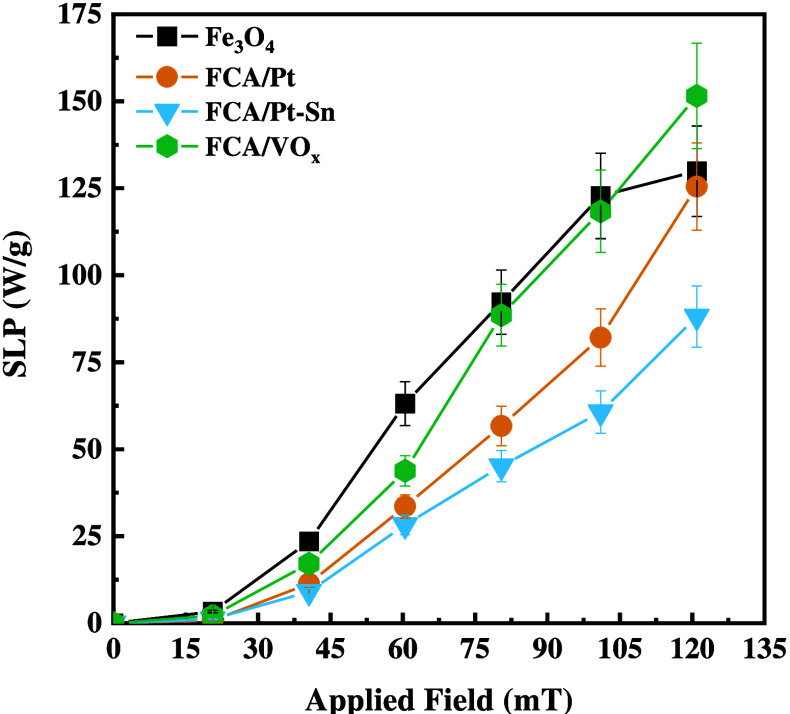
SLP for the starting Fe_3_O_4_ and fresh catalysts (FCA/Pt, FCA/Pt-Sn, and FCA/VO_*x*_) in water (20 mg/mL).

Carbon and Al_2_O_3_ are not ferri- or ferromagnetic; they lack the magnetic hysteresis required for heating. Their addition to magnetic NPs decreases the energy the particles can release on a weight basis. To quantify this decrease, room temperature SLP measurements were performed on Fe_3_O_4_ and the core–shell catalysts and are compared in Figure 6.

All materials showed increasing heat generation as the applied field increased to 121 mT. Generally, Fe_3_O_4_ generated the most heat between 21 and 101 mT. FCA/Pt and FCA/Pt-Sn generally trailed Fe_3_O_4_, although generation by FCA/Pt is nearly equal to that of Fe_3_O_4_ at 121 mT. FCA/VO_*x*_ performed similarly and its generation actually surpassed that of Fe_3_O_4_ at 121 mT. Slight variations in the carbon and Al_2_O_3_ weight percents are not enough to cause large differences in SLP measurements. As discussed earlier and as was shown in the SEM and TEM images, the different amounts of the three Fe phases, the wide size distribution of the Fe_3_O_4_ cores, and the somewhat random assembly of the final catalyst aggregate structures would cause coercivity to vary from batch to batch.

### Catalyst Performance and Analysis of Used Catalysts

Due to the nature of RF-IH and the metallic sheath of the thermocouples, eddy currents generated on them can lead to heat generation that can skew the accuracy of the measurements. Therefore, calibration was performed by operating the RF-IH reactor in the absence of magnetic NPs up to a maximum magnetic field of 247 mT (Figure S9). Above 75 mT, the temperature for the internal bed and external wall thermocouples increased linearly. At the maximum 247 mT, both thermocouples were still reading within 30 °C of each other. However, as shown below, typical applied fields during reaction do not exceed 70 mT, where the difference in the thermocouples is <5 °C. Assuming the error is in the internal bed thermocouple and is related entirely to the applied field, it is clear that, unless other factors intervene when the magnetic NPs are present, no temperature correction is needed in this work. Measurements using the closely related (but more magnetically susceptible) K-thermocouple encased in glass showed only a 22 mK error in within-field versus outside-field measurements at 17 °C and 15.5 mT field strength, and the observed errors *decreased* with respect to temperature.^66^ We added further data supporting the accuracy of our temperature measurements in the Supporting Information.

Before testing the final core–shell catalysts, Fe_3_O_4_@C-1 was tested for catalytic activity in conventional thermal mode at 555 °C. At a WHSV of 2.65 h^–1^ with cofed H_2_, the *n*-butane conversion was below 1.0% with over 90% of the selectivity to C_1_–C_3_ hydrocarbons (Table 3).

**Table 3:** Fe_3_O_4_@C-1 Thermal Operation Reaction Results at 550 °C

		start (0.25 h)			end (5.25 h)		
*n*-butane WHSV (h^–1^)	H_2_/C_4_	conv (%)	selectivity (%)^a^	yield (%)^a^	conv (%)	selectivity (%)^a^	yield (%)^a^
2.65	1.25:1	0.8	5.6	0.05	0.6	4.4	0.03
2.04		4.3	7.7	0.33	4.0	5.9	0.24

a Sum of C_4_H_8_ and C_4_H_6_.

Decreasing the WHSV to 2.04 h^–1^ and feeding no H_2_ increased the activity 4-fold but with selectivity similar to that of cracking products. This is not unexpected because it has been reported that carbons (e.g., coke, carbon nanofibers, and graphitic carbon) can be somewhat active for dehydrogenation and cracking of light alkanes.^3,67,68^

**Figure 7:**
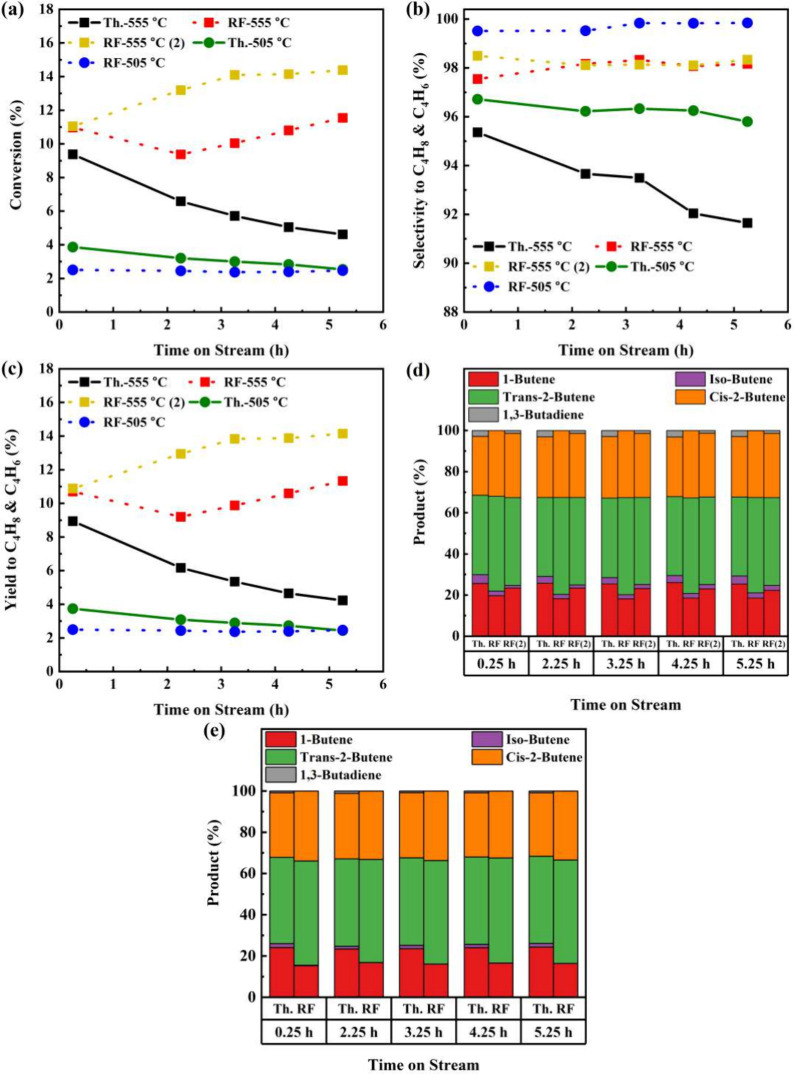
Thermal- and radio-frequency-driven reaction utilizing the FCA/Pt catalyst at 555 and 505 °C (*n*-butane WHSV = 2.65 h^–1^; H_2_:C_4_H_8_ molar ratio = 1.25:1): (a) *n*-butane conversion; (b) selectivity to C_4_H_8_ and C_4_H_6_; (c) yield to C_4_H_8_ and C_4_H_6_; (d) C_4_H_8_ and C_4_H_6_ product distribution at 555 °C; (e) C_4_H_8_ and C_4_H_6_ product distribution at 505 °C.

Starting with FCA/Pt, the activity, selectivity, and yield to butenes and 1,3-butadiene products and the distribution of dehydrogenated 1-butane products are shown in Figure 7 and the temperatures and applied field in Figure S12. The thermal 555 °C (Th.-555 °C) run started with a conversion of 9.4%, but the catalyst deactivated to a roughly constant value by 5.25 h. The selectivity showed a linear decrease throughout the run from 95 to 92%. Switching to RF-IH at 555 °C (RF-555 °C), the initial conversion was 17% (in relative terms) higher than Th.-555 °C and there was a 2.3% (absolute) increase in butenes selectivity. Unexpectedly, the activity increased with respect to TOS, with the selectivity plateauing around 98%. To confirm that this was not an aberration, a second run was performed [RF-555 °C (2)], and a similar trend was observed with conversion also increasing. Therefore, the conversion gap between thermal and radio frequency operation increased because, under conventional thermal heating, the catalyst deactivated. The external wall temperature and applied field increased throughout the RF-IH run (Figure S12b). While this could result from a localized change of the catalyst voidage distribution in the vicinity of the internal thermocouple, the most important takeaway is the absence of deactivation in the RF-IH runs.

At 505 °C, the thermal run’s (Th.-505 °C) activity decreased at a much lower rate compared to Th.-555 °C. Selectivity was very high regardless of the heating method but higher for RF-IH operation. RF-505 °C initially showed lower activity but did not deactivate, with the activity of Th.-505 °C converging to it eventually. The distributions of the products are consistent throughout each run. The RF-IH runs marginally favored the production of 2-butenes, while thermal runs favored more 1-butene, isobutene, and 1,3-butadiene. These distributions show some temperature dependence because at 505 °C the yield to 2-butenes increased.

The average internal bed and external wall temperatures and applied field are listed in Table 4. An immediate observation is the large difference between the internal and external temperatures for RF-IH (167–270 °C), while the difference is much smaller for the thermal run. For RF-505 °C, the average wall temperature is 18–53 °C higher than those of the RF-IH 555 °C runs. This is likely a consequence of uneven catalyst voidage distribution induced by RF-IH.

**Table 4:** Average Internal and External Bed Temperatures and Applied Field Using FCA/Pt (5.25 h Time on Stream)

input temp (°C)	run	avg. internal bed temp (°C)	avg. external wall temp (°C)	avg. RF applied field (mT)
555	thermal	555	553	
	RF-IH	552	282	48
	RF-IH (2)	552	318	49
505	thermal	506	512	
	RF-IH	502	335	36

**Figure 8:**
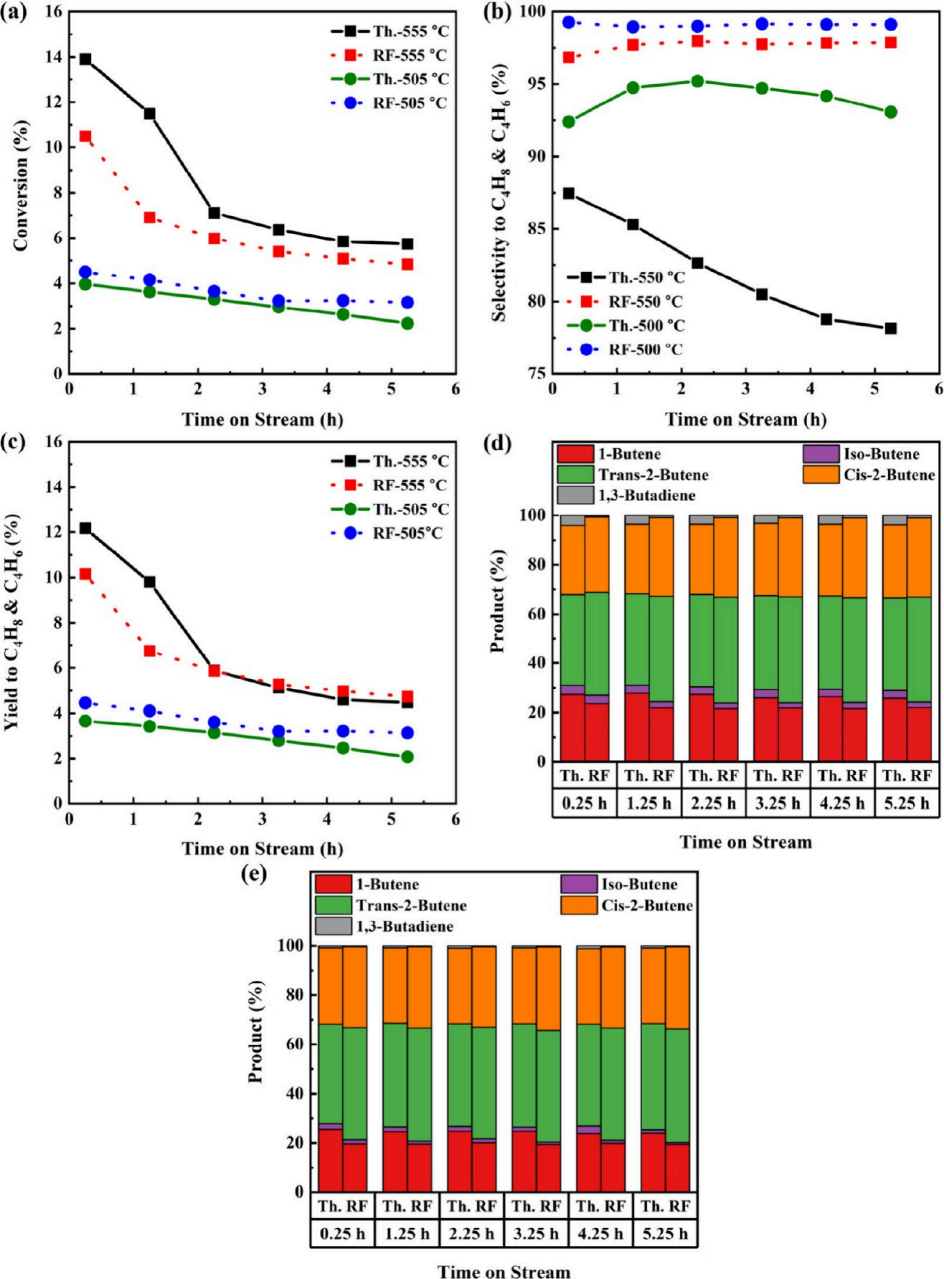
Thermal- and radio-frequency-driven reaction utilizing the FCA/Pt-Sn catalyst at 555 °C (*n*-butane WHSV = 2.65 h^–1^; H_2_:C_4_H_8_ molar ratio = 1.25:1) and 505 °C: (a) *n*-butane conversion; (b) selectivity to C_4_H_8_ and C_4_H_6_: (c) yield to C_4_H_8_ and C_4_H_6_; (d) C_4_H_8_ and C_4_H_6_ product distribution at 550 °C; (e) C_4_H_8_ and C_4_H_6_ product distribution at 505 °C.

The reaction results for FCA/Pt-Sn are shown in Figure 8 and the temperature and applied field data in Figure S13. The Th.-555 °C and RF-555 °C runs showed similar activity behavior. Th.-555 °C began with higher activity, but the difference was greatly reduced by 2.25 h, from 3.4 to 1.1% absolute difference. However, the selectivity for RF-555 °C was vastly improved over the thermal mode of operation. While Th.-555 °C saw a decrease in butenes/butadiene selectivity from 88 to 78% over time, RF-555 °C showed a consistent 97–98%.

At 505 °C, FCA/Pt-Sn followed the same trend in activity as FCA/Pt but with the RF-505 °C run showing both higher activity and less deactivation than Th.-505 °C. For Th.-505 °C, the maximum selectivity was 95%, falling to 93%. In contrast, RF-505 °C showed behavior identical to that of RF-555 °C with a selectivity of 99% throughout the entire run. The FCA/Pt-Sn catalysts operated in the RF-IH mode maintained high and consistent selectivity. The average temperatures and applied field (Table 5) behaved in fashion similar to that of FCA/Pt, again possibly due to uneven catalyst voidage.

**Table 5:** Average Internal and External Bed Temperatures and Applied Field during Dehydrogenation (5.25 h TOS) Using FCA/Pt-Sn

input temp (°C)	run	avg. internal bed temp (°C)	avg. external wall temp (°C)	avg. RF applied field (mT)
555	thermal	555	570	
	RF-IH	552	351	65
505	thermal	507	514	
	RF-IH	502	333	49

**Figure 9:**
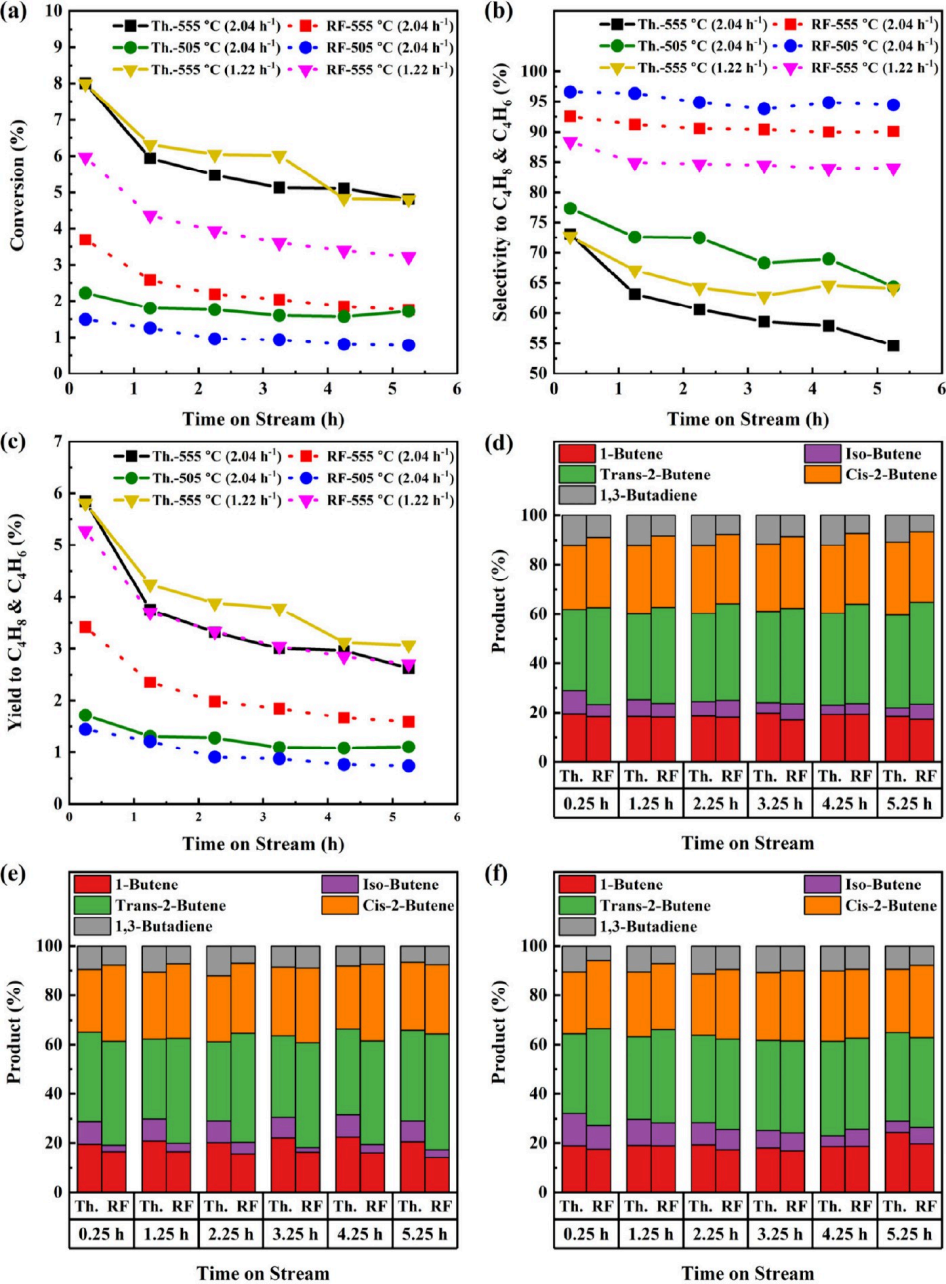
Thermal- and radio-frequency-driven reaction utilizing the FCA/VO_*x*_ catalyst at 555 °C (*n*-butane WHSV = 2.04 h^–1^ and 1.22 h^–1^) and 505 °C (*n*-butane WHSV = 2.04 h^–1^): (a) *n*-butane conversion; (b) selectivity to C_4_H_8_ and C_4_H_6_; (c) yield to C_4_H_8_ and C_4_H_6_; (d) C_4_H_8_ and C_4_H_6_ product distribution at 555 °C (2.04 h^–1^); (e) C_4_H_8_ and C_4_H_6_ product distribution at 505 °C; (f) C_4_H_8_ and C_4_H_6_ product distribution at 555 °C (1.22 h^–1^).

The FCA/VO_*x*_ reaction results are displayed in Figure 9 with the corresponding temperature and applied field data in Figure S14. The thermal and RF-IH 555 °C deactivation behaviors are similar, with the early rate of deactivation being much higher. Compared to RF-555 °C (2.04 h^–1^), Th.-555 °C (2.04 h^–1^) and Th.-555 °C (1.22 h^–1^) are more than twice as active, and compared to RF-555 °C (1.22 h^–1^), they are 34–49% (relative basis) more active. However, while not as active, the RF-IH mode is more selective to butenes/butadiene. Selectivity for RF-555 °C (1.22 h^–1^) does not decrease below 84%, and for RF-555 °C (2.04 h^–1^), it does not decrease below 90%. In contrast, thermal 555 °C (1.22 h^–1^) trends under 70% selectivity and under 60% for Th.-555 °C (2.04 h^–1^).

At 505 °C, FCA/VO_*x*_ follows an activity trend similar to those of the previous two catalysts. Deactivation at this temperature is minimal, but activity is low. Th.-505 °C gives the same poor selectivities as 555 °C, whereas RF-555 °C gives 95–97% C4 alkenes selectivity.

The superior selectivities of FCA/VO_*x*_ RF-555 °C and RF-505 °C over the thermal runs result in yields to butenes/butadiene that are similar. The product distributions matched previous trends found with FCA/Pt and FCA/Pt-Sn, but with minor increases in 1-butene, isobutene, and 1,3-butadiene. At 555 °C, WHSV = 2.04 h^–1^, the thermal run produced more 1,3-butadiene, but isobutene decreased, while it increased for RF-IH. Lowering the temperature to 505 °C caused RF-IH to marginally favor *trans*-2-butene compared with the other runs. Additionally, 1,3-butadiene slowly decreased for the thermal runs, while it slowly increased for RF-IH. At WHSV = 1.22 h^–1^ and 555 °C, the product distribution mirrors the distribution at WHSV = 2.04 h^–1^ but with a modest increase in 1,3-butadiene for both thermal and RF-IH operation. The average temperatures and applied field (Table 6) exhibit behavior similar to those of the other two catalysts.

**Table 6:** Average Internal and External Bed Temperatures and Applied Field during Dehydrogenation (TOS = 5.25 h) Using FCA/VO_*x*_

input temp (°C)	run	avg. internal bed temp (°C)	avg. external wall temp (°C)	avg. RF applied field (mT)
555	thermal (2.04 h^–1^)	555	558	
	RF-IH (2.04 h^–1^)	553	341	43
505	thermal (2.04 h^–1^)	507	500	
	RF-IH (2.04 h^–1^)	503	255	31
555	thermal (1.22 h^–1^)	557	540	-
	RF-IH (1.22 h^–1^)	552	307	51

**Figure 10:**
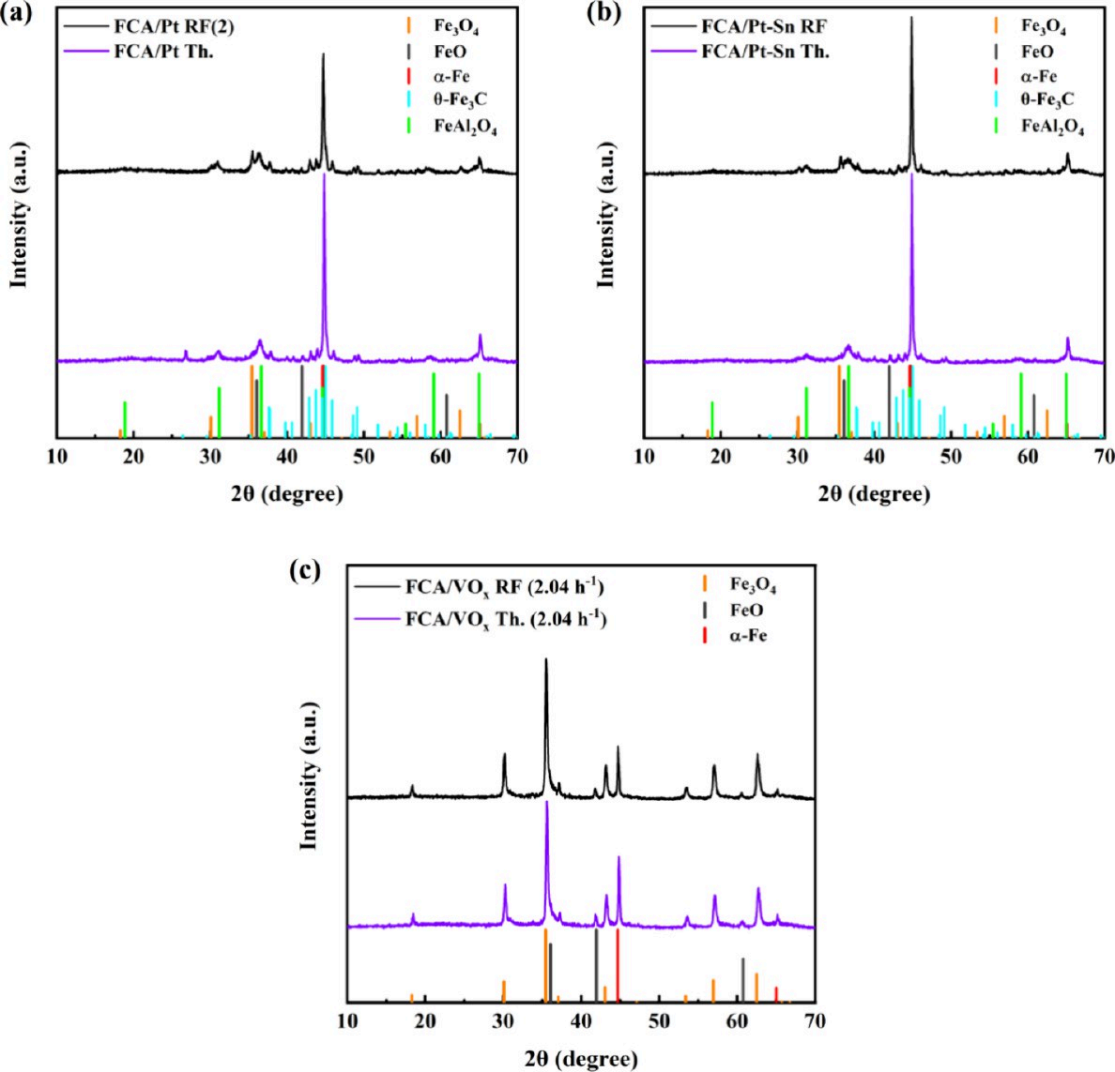
XRD patterns of the spent thermal and RF-IH 555 °C catalysts: (a) FCA/Pt, (b) FCA/Pt-Sn, and (c) FCA/VO_*x*_ (2.04 h^–1^). The drop lines mark the reference patterns.

Postreaction XRD patterns were obtained on three catalysts run at 555 °C: FCA/Pt, FCA/Pt-Sn, and FCA/VO_*x*_, as shown in Figure 10. For both FCA/Pt Th. and FCA/Pt RF(2), most of Fe_3_O_4_ reduced to α-Fe with a little FeO. Furthermore, both thermal and RF-IH catalysts show evidence of θ-Fe_3_C (ICDD: 00-035-0772). Iron carbide formation under these conditions is not unexpected because with carbon present in the shell and coke formation during the reaction, along with cofed H_2_, the reactor environment is similar to that of Fischer–Tropsch, where iron carbide formation takes place.^69,70^ While multiple iron carbide phases exist, for a reactor operating above 350 °C, the formation of the θ phase is favored.^69−72^ Some Fe_3_O_4_ was detected in FCA/Pt RF(2) but not in FCA/Pt Th. Because a significant temperature gradient exists in RF, where the wall is ∼200 °C lower than the catalyst temperature, this could explain the lesser average degree of reduction in FCA/Pt RF(2). Peaks at 30.9°, 36.4°, and 58.7° 2θ were identified as FeAl_2_O_4_ (ICDD: 00-007-0068) in both thermal and radio-frequency used catalysts. Similar phase changes occurred in the FCA/Pt-Sn catalysts. It is evident that it is possible to reduce Fe_3_O_4_ under reaction conditions whether it is located in cores or in the more Al_2_O_3_-rich shells (Figure 5).

FCA/VO_*x*_ (2.04 h^–1^) deviates from the Pt-based catalysts; most of it remained Fe_3_O_4_, with the thermal run used catalyst being reduced slightly more to α-Fe. FeO is present after both thermal and RF-IH operation, but neither showed evidence of iron carbide or FeAl_2_O_4_. Because XRD analysis of the fresh catalysts did not uncover any large variations between FCA/VO_*x*_, FCA/Pt, and FCA/Pt-Sn, it can be concluded that cofeeding H_2_ during the reaction, as is necessary with Pt-based catalysts, causes such catalysts to undergo multiple phase changes under reactor conditions.

**Figure 11:**
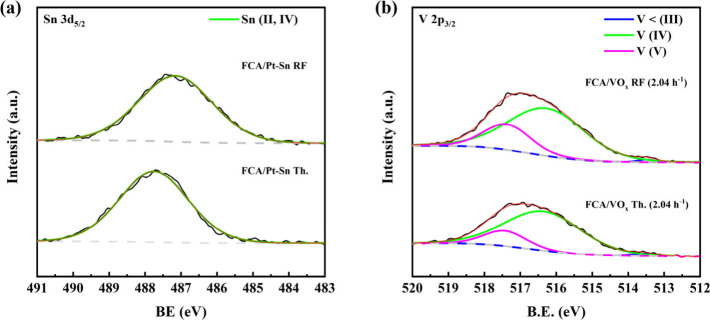
XPS spectra of spent catalysts from thermal and RF-IH 555 °C operation: (a) FCA/Pt-Sn; (b) FCA/VO_*x*_ (2.04 h^–1^).

The Sn 3d_5/2_ XPS spectra (Figure 11a) for the spent 555 °C thermal and RF-IH FCA/Pt-Sn reveal a single peak. The RF-IH Sn peak is at 487.2 eV, with the thermal Sn peak at 487.8 eV. Sn(II,IV)’s binding energy is normally found between 485.8 and 487.6 eV, particularly for Sn in a Pt–Sn/Al_2_O_3_ catalyst.^73−77^ There is no zerovalent Sn (483.5–483.7)^74,76,77^ or Sn as found in true Pt–Sn alloys (∼485.0–485.3 eV).^74,75^ This corresponds to the XANES and XAFS results for the fresh FCA/Pt-Sn, where the majority of Pt appears to be near but not immediately adjacent to Sn. This was further confirmed by overlaying the EDS spectra of Pt and Sn (Figure S15). Sn is almost always at or near a Pt crystallite, although there is evidence of some larger crystallites of Pt, which would have fewer surface atoms and therefore contribute less to the catalysis, that are located further from Sn.

The V surface electronic states for the used catalysts (Figure 11b) are V(<III), V(IV), and V(V). For FCA/VO_*x*_ Th. (2.04 h^–1^), 82% of V is V(IV) with a binding energy of 516.4 eV,^78−83^ while 17.7% is V(V) (517.4 eV). For the FCA/VO_*x*_ RF (2.04 h^–1^), a slight increase in V(V) was observed, 75% V(IV) and 24% V(V), with binding energies of 516.3 and 517.4 eV, respectively. It is typical for a fraction of V(V) to remain after H_2_ reduction and for it to increase over the course of dehydrogenation.^80,82,84,85^ Similar VO_*x*_/γ-Al_2_O_3_ catalysts contained a V(V) fraction between 21 and 35%.^80,82,84−86^ Tian et al.^85^ noted that the V(V) content increased from 23 to 40% after 10 h of isobutane dehydrogenation. However, unlike other V-containing catalysts, FCA/VO_*x*_ appears to lack V(III).^80,82,84,85^ It is possible that the handling procedure (exposure to atmosphere transferring to the XPS) caused oxidation of V(III) to V(IV), which is stable in oxidizing environments.^83,85,86^

Compared to the fresh catalysts, no to little surface area or pore volume was lost during the reactions at 555 °C (Table 7; isotherms plotted in Figure S16). The pore width distributions shifted to slightly larger widths with the exception of FCA/Pt-Sn, where little change took place (Figure S16b). Both thermal and RF-IH used catalysts experienced an increase in carbon due to coking but no increase in hydrogen, suggesting that the coke is highly polyaromatic (Table 7). For the Pt and Pt–Sn catalysts, those used in a RF-IH mode accumulated less coke than their conventionally heated counterparts, and the Pt-only catalyst coked more than its Pt–Sn counterpart. The V-containing catalysts coked the most, except for FCA/VO_*x*_ RF (2.04 h^–1^), which coked in an amount similar to that of FCA/Pt-Sn RF. At the lower space velocity, FCA/VO_*x*_ RF (1.22 h^–1^) accumulated the most coke. It appears that, at WHSV = 1.22 h^–1^, coking is favored over cracking in either thermal or RF-IH modes of operation. Yet, other than at this single condition, FCA/VO_*x*_ coked less when operated in a RF-IH mode.

**Table 7:** Morphological Properties of Fresh and Spent Catalysts, Elemental Analysis, and H_2_ Dispersion Measurements

support/catalyst	surface area (m^2^/g)^a^	pore volume (cm^3^/g)^b^	C (wt %)	H (wt %)	dispersion % (H_2_)
FCA/Pt Fresh	48	0.139	<0.50	<0.50	20
FCA/Pt Th.	47	0.144	1.91	<0.50	19
FCA/Pt RF	52	0.160	1.42	<0.50	28
FCA/Pt RF(2)	53	0.170	1.67	<0.50	15
FCA/Pt-Sn Fresh	79	0.142	1.11	<0.50	12
FCA/Pt-Sn Th.	74	0.148	2.23	<0.50	30
FCA/Pt-Sn RF	75	0.149	2.03	<0.50	7
FCA/VO_*x*_ Fresh	49	0.121	0.53	<0.50	-
FCA/VO_*x*_ Th. (2.04 h^–1^)	42	0.104	2.34	<0.50	-
FCA/VO_*x*_ RF (2.04 h^–1^)	43	0.114	1.15	<0.50	-
FCA/VO_*x*_ Th. (1.22 h^–1^)	42	0.098	2.52	<0.50	-
FCA/VO_*x*_ RF (1.22 h^–1^)	46	0.115	2.58	<0.50	-

a The error is ±10%.

b Mesopores only, no significant micropore volume measured.

As for Pt dispersions (Table 7), the behavior is mixed. No significant dispersion changes were measured for FCA/Pt Th. or FCA/Pt RF. However, for FCA/Pt-Sn, the thermal run saw a 2.5 times increase in dispersion, while the RF-IH run saw about a 40% decrease. In Pt–Sn catalysts, dispersions are sensitive to slight changes in Pt–Sn interactions and segregation, and techniques such as XAFS and TEM are often not able to detect these changes.^87,88^ These results suggest that, for the used Pt–Sn RF-IH catalyst, Pt and Sn interact more upon use, while for the thermal catalyst used, the opposite takes place. While this is not evident in the XPS, it is completely consistent with the results of Figure 8b, which show a large decline in selectivity with respect to TOS for FCA/Pt-Sn Th. 555 °C but not for RF 555 °C. As mentioned in the Introduction, Pt–Sn interaction is critical to high dehydrogenation selectivity and reduces coking. The coking rate can be autocatalytic,^6^ so the prevention of even precursor coke is desireable. For Pt/Al_2_O_3_, it is reported that both coking and the primary reactions slow when the carbon content reaches ∼2 wt %. However, on Pt–Sn/Al_2_O_3_, the coking and primary rates remain constant until ∼7 wt % coke.^6^ This means that Pt–Sn/Al_2_O_3_ can also transfer more coke to the support, extending the catalyst life. Increased migration of coke or its precursors from active metal sites to the support is likely related to electron transfer from Sn to Pt, which as mentioned in the Introduction affects the stability of coke precursors.

SEM images of the used FCA/Pt-Sn Th. 555 °C and FCA/Pt-Sn RF 555 °C catalysts showed no evidence of filamentous or encapsulating coke (Figure S17). This is also true for the HRTEM images (Figure S18). These images also suggest that the carbon/Al_2_O_3_ shells around the Fe/Fe_3_O_4_ NPs were retained during reaction even with extensive reduction of Fe_3_O_4_; see the images and EDS maps for used Pt/FCA in Figure S19. Overlaying Pt EDS with Fe (Figure S20) further demonstrates that most of Pt remains on the outside of the Fe/Fe_3_O_4_ cores in the used catalysts (where Al_2_O_3_ is), although there is some evidence of smaller Pt regions that could be associated more closely with Fe/Fe_3_O_4_.

**Figure 12:**
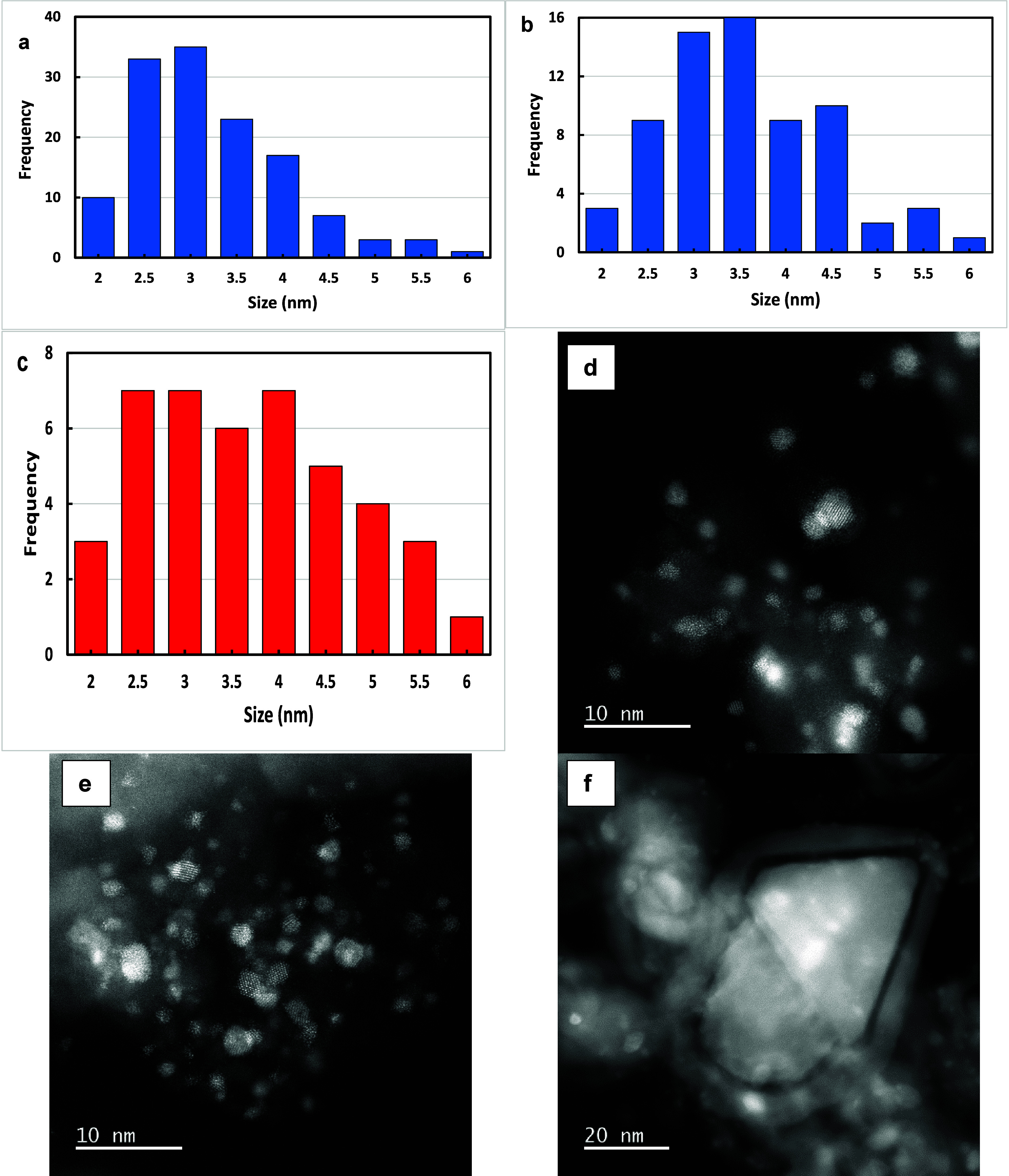
Histograms of size distributions measured from HRTEM images for (a) FCA/Pt-Sn (3.0 nm mean), (b) FCA/Pt (3.3 nm mean), and (c) FCA/Pt after use in the RF-driven reaction (3.4 nm mean). Dark-field images showing Pt crystallites for (d) FCA/Pt-Sn, (e) FCA/Pt, and (f) used FCA/Pt (RF-IH).

We also used the HRTEM images to measure the size distributions of the Pt NPs. Typical images and measured distributions from multiple images are shown in Figure 12 for Pt-containing catalysts. Of note is the similarity in the distributions of both FCA/Pt and FCA/Pt-Sn, and the negligible change in the size distribution of FCA/Pt upon use, ruling out sintering effects as an explanation for any of the changes that took place upon reaction. The used FCA/Pt catalyst EDS maps (Figure S19) do not show any major differences from the fresh catalyst results in Figure 5. There are still regions concentrated in Fe (cores), far more diffuse distributions of Al (shells), and the presence of some Fe outside the cores, with Pt primarily associated with shells.

### Comparisons to Other Work and Their Implications

The synthesis of the catalysts in this work was atypical due to the need for magnetic cores, therefore necessitating the use of precursors and heat treatments that would minimize oxidation of the cores. For this reason, we compare FCA/Pt, FCA/Pt-Sn, and FCA/VO_*x*_ to more typical alkane dehydrogenation catalysts in order to determine their potential value. For comparison purposes, an average rate for the Pt catalysts is calculated by eq 2,
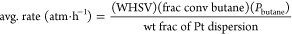
2where this rate would be proportional to the actual average rate for a differential reactor. While the conversions for some runs are inconsistent (>10%) with a true differential reactor, they are not so high that this assumption and metric are without value. The metric allows a comparison of the catalyst activities on a common basis. For V, it is much more difficult to determine the number of active sites for VO_*x*_ because H_2_ or CO chemisorption is not a viable option. Therefore, for the supported VO_*x*_ catalysts, the dispersion is omitted from eq 2. This omission is justified if the V loading does not exceed the theoretical monolayer coverage of the support surface. For a monolayer calculation, we assumed a VO_2.5_ unit area of 0.165 nm^2^;^89,90^ the ratios of total VO_2.5_ area to the surface area of the support are all under 100%.

The calculated average rates increased or remained roughly constant with respect to time for the RF-IH Pt catalyst runs (Table 8), eventually exceeding the rates for thermal operation while maintaining higher selectivity. For the Pt–Sn runs, the thermal run starts with a higher average rate but ends with a rate one-fourth the RF-IH rate. This large variation reflects the increase in dispersion for the thermal run and its decrease for the RF-IH run. With such a large difference in the final dispersions, one might expect the final conversion in a RF-IH mode to be much lower than thermal, but it is not, proving that coking is not responsible for the change in dispersion for the RF-IH catalyst.

**Table 8:** Summary of Catalytic Data, Current Work and Select Publications

catalyst	reaction temp (°C)	WHSV (h^–1^)	feed composition (mol %)	conv (%)	selectivity (%)	avg. rate (atm·h^–1^)	run time (h)	ref^a^
FCA/Pt Th.	555	2.65	H_2_ = 56, nC_4_H_10_ = 44	9.4–4.6	95–92	110–56	5	CW
FCA/Pt RF	555	2.65	H_2_ = 56, nC_4_H_10_ = 44	11–12	98–98	128–100	5	CW
FCA/Pt RF (2)	555	2.65	H_2_ = 56, nC_4_H_10_ = 44	11–14	99–98	128–218	5	CW
FCA/Pt-Sn Th.	555	2.65	H_2_ = 56, nC_4_H_10_ = 44	14–5.7	88–78	272–44	5	CW
FCA/Pt-Sn RF	555	2.65	H_2_ = 56, nC_4_H_10_ = 44	11–4.8	97- 98	214–160	5	CW
FCA/VO_*x*_ Th.	555	2.04	nC_4_H_10_ = 100	8.0–4.8	73–55	5.4–3.3	5	CW
FCA/VO_*x*_ RF	555	2.04	nC_4_H_10_ = 100	3.7–1.8	93–90	2.5–1.2	5	CW
FCA/VO_*x*_ Th.	555	1.22	nC_4_H_10_ = 100	8.0–4.8	73–64	3.3–2.0	5	CW
FCA/VO_*x*_ RF.	555	1.22	nC_4_H_10_ = 100	6.0–3.2	88–84	2.4–1.3	5	CW
Pt (0.3 wt %)/γ-Al_2_O_3_	530	6.0	H_2_ = 56, nC_4_H_10_ = 44	22–10	74–69	255^b^	2	(91)
Pt (0.3 wt %)–Sn (0.35 wt %)/γ-Al_2_O_3_	530	6.0	H_2_ = 56, nC_4_H_10_ = 44	27–10	87–73	1670^b^	2	(91)
Pt (0.5 wt %)/θ-Al_2_O_3_	550	14.8	H_2_ = 34, N_2_ = 34, nC_4_H_10_ = 32	11–8.0	87–85	2084^b^	5	(92)
Pt (0.5 wt %)–Sn (1.0 wt %)/θ-Al_2_O_3_	550	14.8	H_2_ = 34, N_2_ = 34, nC_4_H_10_ = 32	40–31	94–93	2203^b^	5	(92)
VO_*x*_ (2.2 wt %)/γ-Al_2_O_3_	520	2.6	N_2_ = 90, nC_4_H_10_ = 10	8.0–3.7	45–57 (5–20 min)	0.95–0.44	1.2	(69)
VO_*x*_ (3.5 wt %)/θ-Al_2_O_3_	520	27.3	nC_4_H_10_ = 100	33–8.5	56.1^b^	257–266	1	(71)

a CW represents current work.

b Average at the start of the reaction.

Comparing the Pt and Pt–Sn work to that of Bocanegra et al.^91^ and Lee et al.,^92^ the thermal and RF-IH catalysts are in a similar activity range for Pt/γ-Al_2_O_3_, but the ones for Pt or Pt–Sn on θ-Al_2_O_3_ are about 1 order of magnitude more active than the present catalysts. For Bocanegra et al., the measured H_2_ dispersion decreased from 76% to 14% with the addition of Sn, as expected.^6,60^ XPS showed that a fraction of Sn was in the metallic state and likely alloyed with Pt, consistent with the dispersion results.^91^ Lee et al. reported an increase in dispersion from 5 to 17.2% (CO chemisorption), but this is not typical. By HRTEM, the mean Pt particle size was reported to be roughly half the value estimated from the dispersion (12.6 vs 22.5 nm). Possible CO bonding in the bridging mode could lead to the underestimation of dispersion.^93^ However, these are minor considerations because the real advantage of the RF-IH-operated catalysts is in their improved selectivity not their total activity. For both Pt and Pt–Sn catalysts, the selectivity never dropped below 97%, while the best reported selectivity of the other works in Table 8 is 94% by Lee et al. This is particularly notable because the space velocity and alkene selectivity are typically proportional to one another.^3,94^ Operating at the lower space velocities, as here, should have resulted in poorer selectivities, but this was not the case with RF-IH. The selectivity was less than but respectable for thermal FCA/Pt, but the selectivities for the FCA/Pt-Sn thermal run were far worse, below 80%. These results suggest that the RF-IH mode of operation is definitely beneficial for Pt and Pt–Sn dehydrogenation catalysts.

For the V-containing catalysts, the results were also promising. While the thermal runs gave higher conversions, they were much less selective than the RF-IH runs at both space velocities. The average rates for the RF-IH runs at both WHSVs are the same, but not so for the thermal runs, with more coking at lower WHSV. Comparing these results to others’,^90^ both the thermal and RF-IH runs are superior in selectivity and stability. However, work by Jackson and Rugmini^95^ disclosed a more active catalyst, with an average rate 1–2 orders of magnitude higher than that observed here. There are multiple factors that influence the dehydrogenation performance of V-containing catalysts, such as the support type and surface area, V loading, degree of polymerization, and oxidation state. Pinpointing the exact cause of differences in activity is difficult.^3,96^ It is believed that V–O–support moieties are active sites, and the V–O bond catalyzes the C–H bond breaking.^97^ Therefore, the support chemistry is intimately tied to the catalyst performance,^97,98^ and Jackson and Rugmini used a different support than here.^95^ What can be concluded is that while the activity and selectivity of V-containing catalysts trail those of the Pt and Pt–Sn catalysts, RF-IH operation still provides quantifiable improvements in selectivity and catalyst life.

Looking back at the FCA/Pt catalyst, a 20% dispersion corresponds to an average particle size of about 2.0 nm. In the 2.0–4.0 nm range, the (211) and (100) surfaces, which have a lower barrier to deep dehydrogenation but a higher one for alkene desorption, dominate over the (111) surface. It is believed that (211) and (100) surfaces are mainly responsible for hydrogenolysis and coke formation.^3,5,6,99−102^ Below or above this size range is where selectivity improves, and the smaller Pt crystallites also show improved activity,^99−102^ as does atomically dispersed Pt.^8,99,100^ Increases in the particle size well above 2.0 nm, where Pt(111) planes begin to dominate the surface, actually increase propylene selectivity, but it never becomes as high as that of the more dispersed Pt.^99,100^ The increase in performance for the subnanometer clusters is attributed to fewer adjacent Pt–Pt sites; adjacent sites promote C–C bond cleavage,^99^ but they are disrupted by adsorbed or alloyed Sn. Coke (or its precursors) migration to the support limits graphene formation on the metal active sites, prolonging the life of the catalyst.^12,103,104^ However, this migration is thought to occur only for Pt crystallites of <2 nm diameter.^12,103^

Therefore, our higher Pt-based catalyst selectivities and lower rates of deactivation [e.g., FCA/Pt Th. vs FCA/Pt RF and FCA/Pt RF(2)] with RF-IH operation cannot be explained by these “optimal crystallite size” arguments, although the lower activities could. The same catalysts are used under the same conditions, only changing the mode of energy input. Therefore, we conclude that RF-IH operation has some effect on the catalysts, leading to higher alkene selectivities and lower rates of deactivation. One possible explanation (considered further below) is that the RF input affects adsorption of the hydrocarbons and H atoms. Or it is possible that the RF input restructures the Pt surface in a way that is favorable. It is reported that Pt restructuring can sometimes occur during reaction, affecting the coking reactions and hydrogenolysis reactions.^11,103,105,106^ This is a reasonable explanation for our results because the RF-IH operation appears to have an enhanced Pt–Sn interaction, as previously discussed. While the activity for these catalysts lags that of some previous works, the comparisons between thermal and RF-IH modes of operation for the same catalyst show that the lower activity is due to the special requirements of the catalyst synthesis (the Fe_3_O_4_ core and the carbon shell) rather than the mode of energy input.

We reject temperature-specific arguments as the primary cause of the selectivity/stability improvements. As the temperature data (Tables 4–6 and Figures S9–S11) show, the reactor walls during RF-IH operation are much cooler than the center of the catalyst bed, and therefore the catalyst near the walls might be cooler. While the large difference in internal and wall temperatures is probably due to voidage variations inherent in RF-IH operation (one can observe the catalyst moving away from the wall, leaving a gas gap), the temperature gradient nevertheless exists. While such a gradient would benefit alkene selectivity, it must also greatly decrease the overall activity, which was not observed here. Therefore, the hypothesis that the gradient at the wall is somehow responsible for the observed selectivity/stability effects in the RF operation must be rejected.

Selectivity differences arising from RF-IH vs thermal operation have been observed previously. For the RF-IH hydrogenation of oleic acid, we found increased activity and/or selectivity compared to conventional thermal heating.^107^ Mustieles Marin et al.^108^ found that, for hydrodeoxygenation and hydrogenolysis using magnetic FeNi_3_@Ni NPs, the hydrogenolytic cleavage of benzyl phenyl ether was slightly more active and much more selective to toluene and cyclohexanol compared to conventional thermal heating (86 and 88% selectivities vs 64 and 61%), even where the conventionally heated reaction was operated at optimal conditions. Pérez-Camacho et al.^109^ applied RF-IH to dry reforming of methane using a magnetic mixed oxide Na_0.5_La_0.5_Ni_0.3_Al_0.7_O_2.5_. With a packed-bed reactor at 800 °C, they did not observe significant variations in CH_4_ and CO_2_ conversion but found that RF-IH operation reduced carbon formation by a factor of 2.7. When TPO was performed on the spent catalysts, the carbon on the RF-IH catalyst was oxidized fully before reaching 700 °C (in ∼175 min), while it required significantly more time (400 min) to fully oxidize the coke on the thermally operated catalyst. Therefore, RF-IH not only reduced the amount of coking, it also produced less graphitic, easier-to-oxidize coke. It was also found recently that RF-IH increased the activity of Pt/FeO_*x*_ for CO oxidation by 25 times over the conventional thermal mode.^110^

There is a growing belief that an external magnetic field can greatly impact the catalytic performance beyond the elimination of hot spots. Melander et al.^111^ carried out DFT calculations comparing the adsorption and desorption of H_2_ and CO when the magnetic state of FCC (111) Fe is alternated via the magnetic field. Hydrogen readily absorbs on Fe when it is in either its bilayer antiferromagnetic state (AFM2) or its ferromagnetic state (FM). In the AFM2 state, the adsorbed H_2_ spontaneously dissociates, but in the FM state, it forms a stable Kubas complex. The inverse occurs with CO because it is stabilized on the AFM2 surface, while dissociation is much easier on the FM surface. They presented evidence that changing the magnetic state modified the band structure, orbital population, and d-band characteristics. Furthermore, by switching the spin state, charge transfer can also be affected, which, in turn, can affect the stability of the adsorbates. In our work, Fe is not the active site, but adsorption/desorption (of H_2_, for example) on Pt is highly dependent on the charge state and surface structure, and thus these findings are relevant.^111^ Sá et al.^112^ examined a core–shell catalyst similar to this work, Co@C/Pt for CO oxidation. When exposed to a mild magnetic field, magnetized Co modified the Pt electronic state, resulting in a notable (10–14%) decrease in CO oxidation. From *in situ* resonant inelastic X-ray scattering, it appears that changes in the electronic state of Pt, in turn, changed the adsorption geometry because CO in the presence of the field adsorbed more in bridged positions, preventing the necessary migration of O atoms. In summary, there is ample evidence of effects, both positive and negative, of RF-IH on selectivities in catalytic reactions, especially where small molecules such as H_2_ or CO are involved. Furthermore, these previous studies suggest that the improved selectivities and improved Pt–Sn interaction seen in this work are likely due to electronic effects on the active metal.

## Conclusions

This work explored the effects of RF-IH operation on *n*-butane nonoxidative dehydrogenation utilizing magnetically susceptible core–shell catalysts. We have shown that the catalysis of this challenging class of highly endothermic alkane dehydrogenation reactions is feasible with RF-IH energy input, leading to quantifiable benefits, especially on alkene selectivities. After comparable experiments were performed for thermal vs RF-IH heating modes for each of the three typical catalysts, three primary conclusions can be drawn.

(1) For a Pt-based catalyst, the RF-IH catalyst activity improved or remained constant with TOS, at close to 100% selectivity. In contrast, conventional thermal operation resulted in an ∼50% decrease in activity.

(2) While the activity of a thermally operated Pt–Sn catalyst does improve slightly with TOS, this is at the cost of selectivity (88–78%). RF-IH operation results in a noticeable improvement in selectivity, to nearly 100%.

(3) For a V-based catalyst, operation with RF-IH increases selectivity to dehydrogenated products with a marginal impact on activity.

Before and after reaction characterizations of the Pt- and Pt–Sn-based catalysts, employing several surface-sensitive and bulk characterization techniques leads to two other interesting conclusions.

(4) Coking is not responsible for the decrease in Pt–Sn dispersion in RF-IH operation. There is actually an increase in Pt–Sn intimacy during the reaction.

(5) RF-IH also impacts the amount of coke produced, always reducing it for Pt and Pt–Sn catalysts, and mostly so for the VO_*x*_ catalyst.

A literature review revealed some work corroborating these general findings but obtained using simpler reacting or adsorbing systems.

Moving forward, multiple issues in RF-IH catalysis still remain. First, the design of the core–shell magnetically susceptible catalysts might be improved, and because the initial Fe_3_O_4_ cores undergo substantial reduction during alkane dehydrogenation, even with a dense carbon shell, one might consider starting with metallic Fe cores. Second, the issues of optimal Al_2_O_3_ shell thickness and confining Pt to the Al_2_O_3_ shell only were not addressed. Third, the longer-term operation of the catalysts and regeneration methods were not considered. Finally, our results and those of others show that nonthermal (not caused by hot spots) effects on the catalysis exist, and these are possibly related to changes in the electronic states of the active metals that affect the adsorption behavior and surface reconstruction. However, the exact relationships between these effects and magnetic field parameters are largely unknown.

## Supporting Information Available

Preparation details for magnetic NP cores with carbon and Al_2_O_3_ shells, reactor schematics and analytical details, supporting analytical work (XRD, porosimetry, and XAS) for catalyst syntheses, dehydrogenation reactions supporting data (thermocouple calibrations, plots of internal bed and external wall temperatures, and applied fields), and EDS maps, porosimetry, SEM and TEM images of used catalysts (PDF)

## References

[ref1] Weckhuysen B. M.; Schoonheydt R. A. Alkane dehydrogenation over supported chromium oxide catalysts. Catal. Today 1999, 51 (2), 223–232. 10.1016/S0920-5861(99)00047-4.

[ref2] Liu S. B.; Zhang B. F.; Liu G. Z. Metal-based catalysts for the non-oxidative dehydrogenation of light alkanes to light olefins. Reaction Chemistry & Engineering 2021, 6 (1), 9–26. 10.1039/D0RE00381F.

[ref3] Sattler J. J. H. B.; Ruiz-Martinez J.; Santillan-Jimenez E.; Weckhuysen B. M. Catalytic Dehydrogenation of Light Alkanes on Metals and Metal Oxides. Chem. Rev. 2014, 114 (20), 10613–10653. 10.1021/cr5002436. 25163050

[ref4] Otroshchenko T.; Jiang G.; Kondratenko V. A.; Rodemerck U.; Kondratenko E. V. Current status and perspectives in oxidative, non-oxidative and CO2-mediated dehydrogenation of propane and isobutane over metal oxide catalysts. Chem. Soc. Rev. 2021, 50 (1), 473–527. 10.1039/D0CS01140A. 33205797

[ref5] Chen S.; Chang X.; Sun G.; Zhang T.; Xu Y.; Wang Y.; Pei C.; Gong J. Propane dehydrogenation: catalyst development, new chemistry, and emerging technologies. Chem. Soc. Rev. 2021, 50 (5), 3315–3354. 10.1039/D0CS00814A. 33491692

[ref6] Resasco D. E. Dehydrogenation – Heterogeneous. Encyclopedia of Catalysis 2002, 10.1002/0471227617.eoc074.

[ref7] Resasco D. E. Dehydrogenation by Heterogeneous Catalysts. Encyclopedia of Catalysis; Wiley, 2002; pp 49–52. 10.1002/0471227617.eoc074.

[ref8] Saito H.; Sekine Y. Catalytic conversion of ethane to valuable products through non-oxidative dehydrogenation and dehydroaromatization. RSC Adv. 2020, 10 (36), 21427–21453. 10.1039/D0RA03365K. 35518732 PMC9054567

[ref9] Nagaraja B. M.; Shin C.-H.; Jung K.-D. Selective and stable bimetallic PtSn/θ-Al2O3 catalyst for dehydrogenation of n-butane to n-butenes. Applied Catalysis A: General 2013, 467, 211–223. 10.1016/j.apcata.2013.07.022.

[ref10] Virnovskaia A.; Morandi S.; Rytter E.; Ghiotti G.; Olsbye U. Characterization of Pt,Sn/Mg(Al)O Catalysts for Light Alkane Dehydrogenation by FT-IR Spectroscopy and Catalytic Measurements. J. Phys. Chem. C 2007, 111 (40), 14732–14742. 10.1021/jp074686u.

[ref11] Wu J.; Peng Z.; Bell A. T. Effects of composition and metal particle size on ethane dehydrogenation over PtxSn100–x/Mg(Al)O (70⩽x⩽100). J. Catal. 2014, 311, 161–168. 10.1016/j.jcat.2013.11.017.

[ref12] Redekop E. A.; Saerens S.; Galvita V. V.; González I. P.; Sabbe M.; Bliznuk V.; Reyniers M.-F.; Marin G. B. Early stages in the formation and burning of graphene on a Pt/Mg(Al)O dehydrogenation catalyst: A temperature- and time-resolved study. J. Catal. 2016, 344, 482–495. 10.1016/j.jcat.2016.10.023.

[ref13] Fricke C. H.; Bamidele O. H.; Bello M.; Chowdhury J.; Terejanu G.; Heyden A. Modeling the Effect of Surface Platinum–Tin Alloys on Propane Dehydrogenation on Platinum–Tin Catalysts. ACS Catal. 2023, 13 (16), 10627–10640. 10.1021/acscatal.3c00939.

[ref14] Deng L. D.; Miura H.; Shishido T.; Wang Z.; Hosokawa S.; Teramura K.; Tanaka T. Elucidating strong metal-support interactions in Pt-Sn/SiO2 catalyst and its consequences for dehydrogenation of lower alkanes. J. Catal. 2018, 365, 277–291. 10.1016/j.jcat.2018.06.028.

[ref15] Hook A.; Massa J. D.; Celik F. E. Effect of Tin Coverage on Selectivity for Ethane Dehydrogenation over Platinum–Tin Alloys. J. Phys. Chem. C 2016, 120 (48), 27307–27318. 10.1021/acs.jpcc.6b08407.

[ref16] Noble J. P. P.; Bending S. J.; Hill A. K. Radiofrequency Induction Heating for Green Chemicals Manufacture: A Systematic Model of Energy Losses and a Scale-Up Case-Study. ACS Engineering Au 2024, 4 (5), 450–463. 10.1021/acsengineeringau.4c00009. 39429950 PMC11487564

[ref17] Wu L.; Ma H.; Mei J.; Li Y.; Xu Q.; Li Z. Low energy consumption and high quality biofuels production via in-situ fast pyrolysis of reed straw by adding metallic particles in an induction heating reactor. Int. J. Hydrogen Energy 2022, 47, 5828–5841. 10.1016/j.ijhydene.2021.11.229.

[ref18] Li J.; Li J.; Zhu Q.; Li H. Magnetic field acceleration of CO2 reforming of methane over novel hierarchical Co/MgO catalyst in fluidized bed reactor. Chem. Eng. J. 2018, 350, 496–506. 10.1016/j.cej.2018.05.034.

[ref19] Martínez-Prieto L. M.; Marbaix J.; Asensio J. M.; Cerezo-Navarrete C.; Fazzini P.-F.; Soulantica K.; Chaudret B.; Corma A. Ultrastable Magnetic Nanoparticles Encapsulated in Carbon for Magnetically Induced Catalysis. ACS Applied Nano Materials 2020, 3 (7), 7076–7087. 10.1021/acsanm.0c01392. 32743352 PMC7386363

[ref20] Marbaix J.; Mille N.; Lacroix L.-M.; Asensio J. M.; Fazzini P.-F.; Soulantica K.; Carrey J.; Chaudret B. Tuning the Composition of FeCo Nanoparticle Heating Agents for Magnetically Induced Catalysis. ACS Applied Nano Materials 2020, 3 (4), 3767–3778. 10.1021/acsanm.0c00444. PMC7386363 32743352

[ref21] Wang W.; Tuci G.; Duong-Viet C.; Liu Y.; Rossin A.; Luconi L.; Nhut J.-M.; Nguyen-Dinh L.; Pham-Huu C.; Giambastiani G. Induction Heating: An Enabling Technology for the Heat Management in Catalytic Processes. ACS Catal. 2019, 9, 7921–7935. 10.1021/acscatal.9b02471.

[ref22] Sanfilippo D.; Rylander P. N. Hydrogenation and Dehydrogenation. Ullmann’s Encyclopedia of Industrial Chemistry; Wiley, 2009; 10.1002/14356007.a13_487.pub2.

[ref23] Zangeneh F. T.; Taeb A.; Gholivand K.; Sahebdelfar S. Thermodynamic Equilibrium Analysis of Propane Dehydrogenation with Carbon Dioxide and Side Reactions. Chem. Eng. Commun. 2016, 203 (4), 557–565. 10.1080/00986445.2015.1017638.

[ref24] da Silva Moura N.; Bajgiran K. R.; Melvin A. T.; Dooley K. M.; Dorman J. A. Direct Probing of Fe3O4 Nanoparticle Surface Temperatures during Magnetic Heating: Implications for Induction Catalysis. ACS Applied Nano Materials 2021, 4 (12), 13778–13787. 10.1021/acsanm.1c03168.

[ref25] Rosen D. J.; Yang S.; Marino E.; Jiang Z.; Murray C. B. In Situ EXAFS-Based Nanothermometry of Heterodimer Nanocrystals under Induction Heating. J. Phys. Chem. C 2022, 126 (7), 3623–3634. 10.1021/acs.jpcc.2c00608.

[ref26] Pham H. N.; Sattler J. J. H. B.; Weckhuysen B. M.; Datye A. K. Role of Sn in the Regeneration of Pt/γ-Al2O3 Light Alkane Dehydrogenation Catalysts. ACS Catal. 2016, 6 (4), 2257–2264. 10.1021/acscatal.5b02917. 27076991 PMC4822188

[ref27] Abu-Laban M.; Muley P. D.; Hayes D. J.; Boldor D. Ex-situ up-conversion of biomass pyrolysis bio-oil vapors using Pt/Al_2_O_3_ nanostructured catalyst synergistically heated with steel balls via induction. Catal. Today 2017, 291, 3–12. 10.1016/j.cattod.2017.01.010.

[ref28] Mortensen P. M.; Engbæk J. S.; Vendelbo S. B.; Hansen M. F.; Østberg M. Direct hysteresis heating of catalytically active Ni–Co nanoparticles as steam reforming catalyst. Ind. Eng. Chem. Res. 2017, 56 (47), 14006–14013. 10.1021/acs.iecr.7b02331.

[ref29] Varsano F.; Bellusci M.; Provini A.; Petrecca M. NiCo as catalyst for magnetically induced dry reforming of methane. IOP Conference Series: Materials Science and Engineering 2018, 323 (1), 012005 10.1088/1757-899X/323/1/012005.

[ref30] Scarfiello C.; Bellusci M.; Pilloni L.; Pietrogiacomi D.; La Barbera A.; Varsano F. Supported catalysts for induction-heated steam reforming of methane. Int. J. Hydrogen Energy 2021, 46 (1), 134–145. 10.1016/j.ijhydene.2020.09.262.

[ref31] Pham T. T. P.; Ro K. S.; Chen L.; Mahajan D.; Siang T. J.; Ashik U. P. M.; Hayashi J.-i.; Pham Minh D.; Vo D.-V. N. Microwave-assisted dry reforming of methane for syngas production: a review. Environmental Chemistry Letters 2020, 18 (6), 1987–2019. 10.1007/s10311-020-01055-0.

[ref32] Dennis C. L.; Ivkov R. Physics of heat generation using magnetic nanoparticles for hyperthermia. International Journal of Hyperthermia 2013, 29 (8), 715–729. 10.3109/02656736.2013.836758. 24131317

[ref33] Kirschning A.; Kupracz L.; Hartwig J. New synthetic opportunities in miniaturized flow reactors with inductive heating. Chem. Lett. 2012, 41 (6), 562–570. 10.1246/cl.2012.562.

[ref34] Lu A.-H.; Salabas E. L.; Schüth F. Magnetic Nanoparticles: Synthesis, Protection, Functionalization, and Application. Angew. Chem., Int. Ed. 2007, 46 (8), 1222–1244. 10.1002/anie.200602866. 17278160

[ref35] Akbarzadeh A.; Samiei M.; Davaran S. Magnetic nanoparticles: preparation, physical properties, and applications in biomedicine. Nanoscale Res. Lett. 2012, 7 (1), 144–144. 10.1186/1556-276X-7-144. 22348683 PMC3312841

[ref36] Li Q.; Kartikowati C. W.; Horie S.; Ogi T.; Iwaki T.; Okuyama K. Correlation between particle size/domain structure and magnetic properties of highly crystalline Fe3O4 nanoparticles. Sci. Rep 2017, 7 (1), 9894 10.1038/s41598-017-09897-5. 28855564 PMC5577113

[ref37] Houlding T. K.; Rebrov E. V. Application of alternative energy forms in catalytic reactor engineering. Green Processing and Synthesis 2012, 1 (1), 19–31. 10.1515/greenps-2011-0502.

[ref38] Batlle X.; Labarta A. Finite-size effects in fine particles: magnetic and transport properties. J. Phys. D: Appl. Phys. 2002, 35 (6), 201 10.1088/0022-3727/35/6/201.

[ref39] Spaldin N. A. Mangetic Materials: Fundamentals and Applications; Cambridge Universtiy Press, 2011.

[ref40] Bora D. K.; Braun A.; Erat S.; Safonova O.; Graule T.; Constable E. C. Evolution of structural properties of iron oxide nano particles during temperature treatment from 250 °C–900 °C: X-ray diffraction and Fe K-shell pre-edge X-ray absorption study. Curr. Appl. Phys. 2012, 12 (3), 817–825. 10.1016/j.cap.2011.11.013.

[ref41] López-Ortega A.; Estrader M.; Salazar-Alvarez G.; Roca A. G.; Nogués J. Applications of exchange coupled bi-magnetic hard/soft and soft/hard magnetic core/shell nanoparticles. Phys. Rep. 2015, 553, 1–32. 10.1016/j.physrep.2014.09.007.

[ref42] Levy D.; Giustetto R.; Hoser A. Structure of magnetite (Fe3O4) above the Curie temperature: a cation ordering study. Physics and Chemistry of Minerals 2012, 39 (2), 169–176. 10.1007/s00269-011-0472-x.

[ref43] Mohapatra J.; Zeng F.; Elkins K.; Xing M.; Ghimire M.; Yoon S.; Mishra S. R.; Liu J. P. Size-dependent magnetic and inductive heating properties of Fe3O4 nanoparticles: scaling laws across the superparamagnetic size. Phys. Chem. Chem. Phys. 2018, 20 (18), 12879–12887. 10.1039/C7CP08631H. 29700525

[ref44] Almind M. R.; Vendelbo S. B.; Hansen M. F.; Vinum M. G.; Frandsen C.; Mortensen P. M.; Engbæk J. S. Improving performance of induction-heated steam methane reforming. Catal. Today 2020, 342, 13–20. 10.1016/j.cattod.2019.05.005.

[ref45] Kale S. S.; Asensio J. M.; Estrader M.; Werner M.; Bordet A.; Yi D.; Marbaix J.; Fazzini P.-F.; Soulantica K.; Chaudret B. Iron carbide or iron carbide/cobalt nanoparticles for magnetically-induced CO_2_ hydrogenation over Ni/SiRAlOx catalysts. Catalysis Science & Technology 2019, 9 (10), 2601–2607. 10.1039/C9CY00437H.

[ref46] Frey F. E.; Huppke W. F. Equilibrium Dehydrogenation of Ethane, Propane, and the Butanes. Industrial & Engineering Chemistry 1933, 25 (1), 54–59. 10.1021/ie50277a013.

[ref47] Saxena R.; De M. Enhanced performance of supported Pd-Pt bimetallic catalysts prepared by modified electroless deposition for butane dehydrogenation. Appl. Catal. A: General 2021, 610, 117933 10.1016/j.apcata.2020.117933.

[ref48] Matveyeva A. N.; Wärnå J.; Pakhomov N. A.; Murzin D. Y. Kinetic modeling of isobutane dehydrogenation over Ga2O3/Al2O3 catalyst. Chem. Eng. J. 2020, 381, 122741 10.1016/j.cej.2019.122741.

[ref49] Geilen F. M. A.; Stochniol G.; Peitz S.; Schulte-Koerne E. Butenes. Ullmann’s Encyclopedia of Industrial Chemistry; Wiley, 2014; pp 1–13.

[ref50] Mascal M. Chemicals from biobutanol: technologies and markets. Biofuels, Bioproducts and Biorefining 2012, 6 (4), 483–493. 10.1002/bbb.1328.

[ref51] Camacho-Bunquin J.; Ferrandon M. S.; Sohn H.; Kropf A. J.; Yang C.; Wen J.; Hackler R. A.; Liu C.; Celik G.; Marshall C. L.; et al. Atomically Precise Strategy to a PtZn Alloy Nanocluster Catalyst for the Deep Dehydrogenation of n-Butane to 1,3-Butadiene. ACS Catal. 2018, 8 (11), 10058–10063. 10.1021/acscatal.8b02794.

[ref52] Zhang Y.; Qi L.; Leonhardt B.; Bell A. T. Mechanism and Kinetics of n-Butane Dehydrogenation to 1,3-Butadiene Catalyzed by Isolated Pt Sites Grafted onto ≡SiOZn–OH Nests in Dealuminated Zeolite Beta. ACS Catal. 2022, 12 (6), 3333–3345. 10.1021/acscatal.2c00059.

[ref53] Ravel B.; Newville M. ATHENA, ARTEMIS, HEPHAESTUS: data analysis for X-ray absorption spectroscopy using IFEFFIT. Journal of Synchrotron Radiation 2005, 12 (4), 537–541. 10.1107/S0909049505012719. 15968136

[ref54] Roman C. L. Induction Heating Driven Heterogeneous Catalysis: Magnetically Induced Nanoparticle Catalysts. Doctoral Dissertation, Louisiana State University, Baton Rouge, LA, 2023.

[ref55] Geng J.; Jefferson D. A.; Johnson B. F. G. Direct conversion of iron stearate into magnetic Fe and Fe3C nanocrystals encapsulated in polyhedral graphite cages. Chem. Commun. 2004, (21), 2442–2443. 10.1039/b406227b. 15514806

[ref56] Lak A.; Kraken M.; Ludwig F.; Kornowski A.; Eberbeck D.; Sievers S.; Litterst F. J.; Weller H.; Schilling M. Size dependent structural and magnetic properties of FeO–Fe3O4 nanoparticles. Nanoscale 2013, 5 (24), 12286–12295. 10.1039/c3nr04562e. 24154669

[ref57] Sawatzki S.; Heller R.; Mickel C.; Seifert M.; Schultz L.; Neu V. Largely enhanced energy density in epitaxial SmCo5/Fe/SmCo5 exchange spring trilayers. J. Appl. Phys. 2011, 109 (12), 123922 10.1063/1.3596756.

[ref58] Klemmer T.; Hoydick D.; Okumura H.; Zhang B.; Soffa W. A. Magnetic hardening and coercivity mechanisms in L10 ordered FePd ferromagnets. Scripta Metallurgica et Materialia 1995, 33 (10), 1793–1805. 10.1016/0956-716X(95)00413-P.

[ref59] Wu Z.; Kim H.-S.; Stair P. C.; Rugmini S.; Jackson S. D. On the Structure of Vanadium Oxide Supported on Aluminas: UV and Visible Raman Spectroscopy, UV–Visible Diffuse Reflectance Spectroscopy, and Temperature-Programmed Reduction Studies. J. Phys. Chem. B 2005, 109 (7), 2793–2800. 10.1021/jp046011m. 16851289

[ref60] Stagg S. M.; Querini C. A.; Alvarez W. E.; Resasco D. E. Isobutane Dehydrogenation on Pt–Sn/SiO2Catalysts: Effect of Preparation Variables and Regeneration Treatments. J. Catal. 1997, 168 (1), 75–94. 10.1006/jcat.1997.1617.

[ref61] Beale A. M.; Weckhuysen B. M. EXAFS as a tool to interrogate the size and shape of mono and bimetallic catalyst nanoparticles. Phys. Chem. Chem. Phys. 2010, 12 (21), 5562–5574. 10.1039/b925206a. 20379576

[ref62] Golas P. L.; Louie S.; Lowry G. V.; Matyjaszewski K.; Tilton R. D. Comparative Study of Polymeric Stabilizers for Magnetite Nanoparticles Using ATRP. Langmuir 2010, 26 (22), 16890–16900. 10.1021/la103098q. 20945936

[ref63] Yeap S. P.; Lim J.; Ooi B. S.; Ahmad A. L. Agglomeration, colloidal stability, and magnetic separation of magnetic nanoparticles: collective influences on environmental engineering applications. J. Nanopart. Res. 2017, 19 (11), 368 10.1007/s11051-017-4065-6.

[ref64] Mahendra I.; Linh M.; Thang N.; Thuy V.; Trang L.; Thinh L.; Phuong N.; Ha N.; Thuong N.; Kawahara S.; et al. Protein Removal from Natural Rubber Latex with Fe_3_O_4_@Al_2_O_3_ Nanoparticle. J. Braz. Chem. Soc. 2021, 10.21577/0103-5053.20200182.

[ref65] Ammar S. H.; Ibrahim Elaibi A.; Mohammed I. S. Core/shell Fe_3_O_4_@Al_2_O_3_-PMo magnetic nanocatalyst for photocatalytic degradation of organic pollutants in an internal loop airlift reactor. J. Water Process Eng. 2020, 37, 101240 10.1016/j.jwpe.2020.101240.

[ref66] Begus S.; Bojkovski J.; Drnovsek J.; Gersak G. Magnetic effects on thermocouples. Meas. Sci. Technol. 2014, 25, 035006 10.1088/0957-0233/25/3/035006.

[ref67] McGregor J.; Huang Z.; Parrott E. P. J.; Zeitler J. A.; Nguyen K. L.; Rawson J. M.; Carley A.; Hansen T. W.; Tessonnier J.-P.; Su D. S.; et al. Active coke: Carbonaceous materials as catalysts for alkane dehydrogenation. J. Catal. 2010, 269 (2), 329–339. 10.1016/j.jcat.2009.11.016.

[ref68] Wang R.; Sun X.; Zhang B.; Sun X.; Su D. Hybrid Nanocarbon as a Catalyst for Direct Dehydrogenation of Propane: Formation of an Active and Selective Core–Shell sp2/sp3 Nanocomposite Structure. Chem. Eur. J. 2014, 20 (21), 6324–6331. 10.1002/chem.201400018. 24740731

[ref69] de Smit E.; Cinquini F.; Beale A. M.; Safonova O. V.; van Beek W.; Sautet P.; Weckhuysen B. M. Stability and Reactivity of ϵ−χ–θ Iron Carbide Catalyst Phases in Fischer–Tropsch Synthesis: Controlling μC. J. Am. Chem. Soc. 2010, 132 (42), 14928–14941. 10.1021/ja105853q. 20925335

[ref70] Chang Q.; Zhang C.; Liu C.; Wei Y.; Cheruvathur A. V.; Dugulan A. I.; Niemantsverdriet J. W.; Liu X.; He Y.; Qing M.; et al. Relationship between Iron Carbide Phases (ε-Fe2C, Fe7C3, and χ-Fe5C2) and Catalytic Performances of Fe/SiO2 Fischer–Tropsch Catalysts. ACS Catal. 2018, 8 (4), 3304–3316. 10.1021/acscatal.7b04085.

[ref71] Liu X.-W.; Zhao S.; Meng Y.; Peng Q.; Dearden A. K.; Huo C.-F.; Yang Y.; Li Y.-W.; Wen X.-D. Mössbauer Spectroscopy of Iron Carbides: From Prediction to Experimental Confirmation. Sci. Rep. 2016, 6 (1), 26184 10.1038/srep26184. 27189083 PMC4870625

[ref72] Andersson S.; Hyde B. G. Twinning on the unit cell level as a structure-building operation in the solid state. J. Solid State Chem. 1974, 9 (1), 92–101. 10.1016/0022-4596(74)90059-0.

[ref73] Ballarini A. D.; Zgolicz P.; Vilella I. M. J.; de Miguel S. R.; Castro A. A.; Scelza O. A. n-Butane dehydrogenation on Pt, PtSn and PtGe supported on γ-Al2O3 deposited on spheres of α-Al2O3 by washcoating. Applied Catalysis A: General 2010, 381 (1), 83–91. 10.1016/j.apcata.2010.03.053.

[ref74] Bocanegra S. A.; de Miguel S. R.; Borbath I.; Margitfalvi J. L.; Scelza O. A. Behavior of bimetallic PtSn/Al2O3 catalysts prepared by controlled surface reactions in the selective dehydrogenation of butane. J. Mol. Catal. a-Chem. 2009, 301 (1–2), 52–60. 10.1016/j.molcata.2008.11.006.

[ref75] Seo H.; Lee J. K.; Hong U. G.; Park G.; Yoo Y.; Lee J.; Chang H.; Song I. K. Direct dehydrogenation of n-butane over Pt/Sn/M/γ-Al2O3 catalysts: Effect of third metal (M) addition. Catal. Commun. 2014, 47, 22–27. 10.1016/j.catcom.2014.01.007.

[ref76] de Miguel S.; Castro A.; Scelza O.; Fierro J. L. G.; Soria J. FTIR and XPS study of supported PtSn catalysts used for light paraffins dehydrogenation. Catal. Lett. 1996, 36 (3), 201–206. 10.1007/BF00807620.

[ref77] Balakrishnan K.; Schwank J. A chemisorption and XPS study of bimetallic Pt-Sn/Al2O3 catalysts. J. Catal. 1991, 127 (1), 287–306. 10.1016/0021-9517(91)90227-U.

[ref78] Biesinger M. C.; Lau L. W. M.; Gerson A. R.; Smart R. S. C. Resolving surface chemical states in XPS analysis of first row transition metals, oxides and hydroxides: Sc, Ti, V, Cu and Zn. Appl. Surf. Sci. 2010, 257 (3), 887–898. 10.1016/j.apsusc.2010.07.086.

[ref79] Silversmit G.; Depla D.; Poelman H.; Marin G. B.; De Gryse R. Determination of the V2p XPS binding energies for different vanadium oxidation states (V5+ to V0+). J. Electron Spectrosc. Relat. Phenom. 2004, 135 (2), 167–175. 10.1016/j.elspec.2004.03.004.

[ref80] Harlin M. E.; Niemi V. M.; Krause A. O. I. Alumina-Supported Vanadium Oxide in the Dehydrogenation of Butanes. J. Catal. 2000, 195 (1), 67–78. 10.1006/jcat.2000.2969.

[ref81] Liu G.; Zhao Z.-J.; Wu T.; Zeng L.; Gong J. Nature of the Active Sites of VOx/Al2O3 Catalysts for Propane Dehydrogenation. ACS Catal. 2016, 6 (8), 5207–5214. 10.1021/acscatal.6b00893.

[ref82] Shan Y.-L.; Sun H.-L.; Zhao S.-L.; Li K.-X.; Xia K.-H.; Ding J.-W.; Yu W.-L. Enhancing propane direct dehydrogenation performances through temperature induced VOx dispersion and alumina support phase transformation. Chemical Engineering Journal 2022, 450, 137969 10.1016/j.cej.2022.137969.

[ref83] Bai P.; Ma Z.; Li T.; Tian Y.; Zhang Z.; Zhong Z.; Xing W.; Wu P.; Liu X.; Yan Z. Relationship between Surface Chemistry and Catalytic Performance of Mesoporous γ-Al2O3 Supported VOX Catalyst in Catalytic Dehydrogenation of Propane. ACS Appl. Mater. Interfaces 2016, 8 (39), 25979–25990. 10.1021/acsami.6b07779. 27636162

[ref84] Shan Y.-L.; Zhao W.-T.; Zhao S.-L.; Wang X.-X.; Sun H.-L.; Yu W.-L.; Ding J.-W.; Feng X.; Chen D. Effects of alumina phases on the structure and performance of VOx/Al2O3 catalysts in non-oxidative propane dehydrogenation. Molecular Catalysis 2021, 504, 111466 10.1016/j.mcat.2021.111466.

[ref85] Tian Y.-P.; Bai P.; Liu S.-M.; Liu X.-M.; Yan Z.-F. VOx–K2O/γ-Al2O3 catalyst for nonoxidative dehydrogenation of isobutane. Fuel Process. Technol. 2016, 151, 31–39. 10.1016/j.fuproc.2016.05.024.

[ref86] Yang Q.-Q.; Hu P.; Xiu N.-Y.; Lang W.-Z.; Guo Y.-J. VOx/γ-Al2O3 Catalysts for Propane Dehydrogenation Prepared by “Impregnation-Solid Phase Reaction” Method with Aluminum Hydroxide as Support Precursor. ChemistrySelect 2018, 3 (35), 10049–10055. 10.1002/slct.201802070.

[ref87] Liu L.; Lopez-Haro M.; Lopes C. W.; Rojas-Buzo S.; Concepcion P.; Manzorro R.; Simonelli L.; Sattler A.; Serna P.; Calvino J. J.; et al. Structural modulation and direct measurement of subnanometric bimetallic PtSn clusters confined in zeolites. Nature Catalysis 2020, 3 (8), 628–638. 10.1038/s41929-020-0472-7.

[ref88] Sattler A.; Paccagnini M.; Liu L.; Gomez E.; Klutse H.; Burton A. W.; Corma A. Assessment of metal-metal interactions and catalytic behavior in platinum-tin bimetallic subnanometric clusters by using reactive characterizations. J. Catal. 2021, 404, 393–399. 10.1016/j.jcat.2021.10.006.

[ref89] Eon J. G.; Olier R.; Volta J. C. Oxidative Dehydrogenation of Propane on γ-Al2O3 Supported Vanadium Oxides. J. Catal. 1994, 145 (2), 318–326. 10.1006/jcat.1994.1040.

[ref90] Volpe M.; Tonetto G.; de Lasa H. Butane dehydrogenation on vanadium supported catalysts under oxygen free atmosphere. Applied Catalysis A: General 2004, 272 (1), 69–78. 10.1016/j.apcata.2004.05.017.

[ref91] Bocanegra S.; Ballarini A.; Zgolicz P.; Scelza O.; de Miguel S. Highly selective and stable bimetallic catalysts supported on different materials for n-butane dehydrogenation. Catal. Today 2009, 143 (3), 334–340. 10.1016/j.cattod.2008.10.002.

[ref92] Lee M.-H.; Nagaraja B. M.; Lee K. Y.; Jung K.-D. Dehydrogenation of alkane to light olefin over PtSn/θ-Al2O3 catalyst: Effects of Sn loading. Catal. Today 2014, 232, 53–62. 10.1016/j.cattod.2013.10.011.

[ref93] Kalhara Gunasooriya G. T. K.; Saeys M. CO Adsorption Site Preference on Platinum: Charge Is the Essence. ACS Catal. 2018, 8 (5), 3770–3774. 10.1021/acscatal.8b00214.

[ref94] Yang H.; Xu L.; Ji D.; Wang Q.; Lin L. The Catalytic Dehydrogenation of c2h6 to c2h4 under non-Oxidative Conditions over the 6cr/g-al2o3Catalyst. React. Kinet. Catal. Lett. 2002, 76 (1), 151–159. 10.1023/A:1015633915938.

[ref95] Jackson S. D.; Rugmini S. Dehydrogenation of n-butane over vanadia catalysts supported on θ-alumina. J. Catal. 2007, 251 (1), 59–68. 10.1016/j.jcat.2007.07.015.

[ref96] Weckhuysen B. M.; Keller D. E. Chemistry, spectroscopy and the role of supported vanadium oxides in heterogeneous catalysis. Catal. Today 2003, 78 (1), 25–46. 10.1016/S0920-5861(02)00323-1.

[ref97] Keller D. E.; Airaksinen S. M. K.; Krause A. O.; Weckhuysen B. M.; Koningsberger D. C. Atomic XAFS as a Tool To Probe the Reactivity of Metal Oxide Catalysts: Quantifying Metal Oxide Support Effects. J. Am. Chem. Soc. 2007, 129 (11), 3189–3197. 10.1021/ja0667007. 17323947

[ref98] Carter J. H.; Bere T.; Pitchers J. R.; Hewes D. G.; Vandegehuchte B. D.; Kiely C. J.; Taylor S. H.; Hutchings G. J. Direct and oxidative dehydrogenation of propane: from catalyst design to industrial application. Green Chem. 2021, 23 (24), 9747–9799. 10.1039/D1GC03700E.

[ref99] Zhang W.; Wang H.; Jiang J.; Sui Z.; Zhu Y.; Chen D.; Zhou X. Size Dependence of Pt Catalysts for Propane Dehydrogenation: from Atomically Dispersed to Nanoparticles. ACS Catal. 2020, 10 (21), 12932–12942. 10.1021/acscatal.0c03286.

[ref100] Zhu J.; Yang M.-L.; Yu Y.; Zhu Y.-A.; Sui Z.-J.; Zhou X.-G.; Holmen A.; Chen D. Size-Dependent Reaction Mechanism and Kinetics for Propane Dehydrogenation over Pt Catalysts. ACS Catal. 2015, 5 (11), 6310–6319. 10.1021/acscatal.5b01423.

[ref101] Yang M.-L.; Zhu Y.-A.; Fan C.; Sui Z.-J.; Chen D.; Zhou X.-G. DFT study of propane dehydrogenation on Pt catalyst: effects of step sites. Phys. Chem. Chem. Phys. 2011, 13 (8), 3257–3267. 10.1039/c0cp00341g. 21253636

[ref102] Yang M.-L.; Zhu J.; Zhu Y.-A.; Sui Z.-J.; Yu Y.-D.; Zhou X.-G.; Chen D. Tuning selectivity and stability in propane dehydrogenation by shaping Pt particles: A combined experimental and DFT study. J. Mol. Catal. A: Chem. 2014, 395, 329–336. 10.1016/j.molcata.2014.08.008.

[ref103] Peng Z.; Somodi F.; Helveg S.; Kisielowski C.; Specht P.; Bell A. T. High-resolution in situ and ex situ TEM studies on graphene formation and growth on Pt nanoparticles. J. Catal. 2012, 286, 22–29. 10.1016/j.jcat.2011.10.008.

[ref104] Srihiranpullop S.; Praserthdam P.; Mongkhonsi T. Deactivation of the metal and acidic functions for Pt, Pt-Sn and Pt-Sn-K using physically mixed catalysts. Korean Journal of Chemical Engineering 2000, 17 (5), 548–552. 10.1007/BF02707164.

[ref105] Horch S.; Lorensen H. T.; Helveg S.; Lægsgaard E.; Stensgaard I.; Jacobsen K. W.; Nørskov J. K.; Besenbacher F. Enhancement of surface self-diffusion of platinum atoms by adsorbed hydrogen. Nature 1999, 398 (6723), 134–136. 10.1038/18185.

[ref106] Garnett E. C.; Liang W.; Yang P. Growth and Electrical Characteristics of Platinum-Nanoparticle-Catalyzed Silicon Nanowires. Adv. Mater. 2007, 19 (19), 2946–2950. 10.1002/adma.200700288.

[ref107] Roman C. L.; da Silva Moura N.; Wicker S.; Dooley K. M.; Dorman J. A. Induction Heating of Magnetically Susceptible Nanoparticles for Enhanced Hydrogenation of Oleic Acid. ACS Applied Nano Materials 2022, 5 (3), 3676–3685. 10.1021/acsanm.1c04351. 35372795 PMC8961733

[ref108] Mustieles Marin I.; De Masi D.; Lacroix L.-M.; Fazzini P.-F.; van Leeuwen P. W. N. M.; Asensio J. M.; Chaudret B. Hydrodeoxygenation and hydrogenolysis of biomass-based materials using FeNi catalysts and magnetic induction. Green Chem. 2021, 23 (5), 2025–2036. 10.1039/D0GC03495A.

[ref109] Pérez-Camacho M. N.; Abu-Dahrieh J.; Rooney D.; Sun K. Biogas reforming using renewable wind energy and induction heating. Catal. Today 2015, 242, 129–138. 10.1016/j.cattod.2014.06.010.

[ref110] Adogwa A.; Chukwu E.; Malaj A.; Punyapu V. R.; Chamness O.; Glisson N.; Bruce B.; Lee S.; Zachman M. J.; Bruce D. A.; et al. Catalytic Reaction Triggered by Magnetic Induction HeatingMechanistically Distinguishes Itself from the Standard Thermal Reaction. ACS Catal. 2024, 14 (6), 4008–4017. 10.1021/acscatal.3c05989.

[ref111] Melander M.; Laasonen K.; Jónsson H. Effect of Magnetic States on the Reactivity of an FCC(111) Iron Surface. J. Phys. Chem. C 2014, 118 (29), 15863–15873. 10.1021/jp504709d.

[ref112] Sá J.; Szlachetko J.; Sikora M.; Kavčič M.; Safonova O. V.; Nachtegaal M. Magnetic manipulation of molecules on a non-magnetic catalytic surface. Nanoscale 2013, 5 (18), 8462–8465. 10.1039/c3nr02237d. 23842714

