# Supplementary material for: Improved Performance of Catalysts Containing Pt, Pt–Sn, and V in the Dehydrogenation of n-Butane by Radio-Frequency Induction Heating
Source: ACS Sustain Chem Eng. 2025 Feb 11;13(7):2978–97. doi: 10.1021/acssuschemeng.4c10045 (PMC11863546; doi:10.1021/acssuschemeng.4c10045)
Supplement: Supplementary file 1 — sc4c10045_si_001.pdf [file sc4c10045_si_001.pdf]

Supporting Information - Improved Performance of Catalysts Containing Pt, Pt-Sn and V in the Dehydrogenation of n-Butane by Radio-Frequency Induction Heating

Cameron L. Roman, Jonathan Lucas, James A. Dorman,* and Kerry M. Dooley*

Department of Chemical Engineering, Louisiana State University,

Baton Rouge, LA 70803, United States

*Corresponding authors. Email: jadorman@gmail.com, dooley@lsu.edu

20 Pages

20 Figures

Table of Contents

Preparation Details for Magnetic Nanoparticle Cores with Carbon and Al_2O_3 Shells S3
Reactor Schematics and Analytical Details S4

Figure S1. Dehydrogenation reactor schematic, RF-IH setup shown S4

Figure S2. Pictures/schematics of the thermal and RF-IH reactor setup: (a) catalyst bed with internal thermocouple, (b) complete thermal reactor setup, (c) complete RF-IH reactor setup, (d) closeup of catalyst bed showing the coil and external thermocouple, (e) schematic of thermocouple placement. S5

Figure S3. Chromatogram of a typical separation, dehydrogenation of n-butane over Pt or Pt-Sn catalyst S6

Figure S4. Chromatogram of a typical separation, dehydrogenation of n-butane over VO_x catalyst S7

Supporting Analytical Work for Catalyst Syntheses S8

Figure S5. XRDs of: (a) initial Fe_3O_4 and Fe_3O_4@C core-shell supports; and (b) Fe_3O_4@C@γ-Al_2O_3 ....S8

Figure S6. For the supports and the core-shell catalysts: (a) isotherm linear plots; and (b) pore diameter distributions..S8

Figure S7. Pt L3 Edge (a) XANES and (b) XAFS of FCA/Pt-Sn S9

Figure S8. TEM images of core (Fe_3O_4)-shell (Al_2O_3) syntheses: (a) low magnification FCA-2; (b) low magnification FCA-3; (c, d, e) higher magnifications of FCA-3 with Fe_3O_4 average sizes (darker regions) of 63, 48, and 27 nm respectively...S10

Dehydrogenation Reactions Supporting Data S11

Figure S9. Internal bed and external wall thermocouple temperature calibrations up to the maximum possible applied field (247 mT) S11

Figure S10: Thermocouple susceptibility measurements with 0.75 g Fe_3O_4. "TC below" means the thermocouple was placed below the bed, in the quartz wool. S13

Figure S11: Thermocouple susceptbility measurements with 0.35 g Fe. "TC bottom" means the thermocouple was placed at the exact bottom of the bed, just above the quartz wool. S13

Figure S12. FCA/Pt internal bed and external wall temperatures, and applied field data for the thermal and RF-IH catalysts: (a) reduction of the 555 °C catalyst; (b) dehydrogenation at 555 °C; (c) reduction of the 505 °C catalyst; and (d) dehydrogenation at 505 °C S15

Figure S13. FCA/Pt-Sn Internal Bed, External Wall, and Applied Field data for the Thermal and RF-IH catalysts: (a) reduction of 555 °C catalyst; (b) dehydrogenation at 555 °C; (c) reduction of the 505 °C catalyst; and (d) dehydrogenation at 505 °C S16

Figure S14. FCA/VO_x Internal Bed, External Wall, and Applied Field data for the Thermal and RF-IH catalysts: (a) reduction of 555 °C catalyst (2.04 h^{-1}); (b) dehydrogenation at 555 °C (2.04 h^{-1}) (c) reduction of 505 °C catalyst (2.04 h^{-1}); (d) dehydrogenation at 505 °C (2.04 h^{-1}); (e) reduction of 555 °C catalyst (1.22 h^{-1}); and (f) dehydrogenation at 555 °C (1.22 h^{-1})....... S17

EDS maps, porosimetry, SEMs and TEMs of used catalysts S18

Figure S15. EDS maps of FCA/Pt-Sn, Sn (red) overlaid on Pt (green) S18

Figure S16. For the used catalysts: (a) isotherm linear plots; and (b) pore diameter distributions distributions S19

Figure S17. SEMs of used catalysts: (a) 550 °C FCA/Pt-Sn Th.; (b) same at higher magnification; (c) 550 °C FCA/Pt-Sn RF; and (d) same at higher magnification S19

Figure S18. TEM images of used: (a, b) Pt/FCA-Th.; (c, d) Pt/FCA-RF S20

Figure S19. Scanned electron images and EDS maps of used FCA/Pt-RF (a-d) and used FCA/Pt-Th. (e-h) catalysts: (a, e) scanned electron images; (b, f) Fe EDS; (c, g) Al EDS; (d, h) Pt EDS S21

Figure S20. EDS maps of used FCA/Pt, Pt (green) overlaid on Fe (red): (a-c) FCA/Pt-RF; (d-f) FCA/Pt-Th S22

References S22

Preparation Details for Magnetic Nanoparticle Cores with Carbon and Al_2O_3 Shells

Fe_3O_4 nanoparticles (NPs, Alfa Aesar, 97%, 50-100 nm 8.5 – 11.5 m^2/g) were the catalyst cores. Sucrose polyol was the carbon source for the carbon shell. Aluminum isopropoxide (AIP, MCB, tech grade) and ethanol (ACS Certified, 95% EtOH, 5% MeOH) were used in Al_2O_3 shell deposition. Tetraamine-platinum (II) nitrate (Aesar, 99.6%), tetrabutyltin (Aldrich, tech grade 93%), and pentane (Fisher, ACS Certified) were used for Pt and Pt-Sn impregnation while purified ammonium vanadate (Fisher, 99.5%) and oxalic acid (Baker and Adamson, reagent grade) were used for vanadium impregnation. Hydrochloric acid (HCl, 36.5−38%, VWR) and nitric acid (HNO_3, 68−70%, VWR) were used for sample digestion prior to inductively coupled plasma optical emission spectrometry (ICP-OES).

The Fe_3O_4@carbon core-shell magnetic NPs were prepared via carbonization of sucrose polyol, twice, to give a nominal Fe:C molar ratio of 1:10. Two batches were prepared: one for Pt- and Pt-Sn-containing catalysts (Fe_3O_4@C-1) and one for a VO_x-based catalyst (Fe_3O_4@C-2). The heat-treated (500 °C 1 h, 5% H_2) Fe_3O_4 NPs were mixed with equal weights of sucrose polyol and and 3.0 wt% H_2SO_4 for 1.5 h at room temperature, then dried at 120 °C for 24 h and carbonized at 400 °C (5 °C min^{-1}) for 3 h, then ramped (5 °C min^{-1}) to 850 °C for an additional 3 h in N_2. The procedure was then repeated.

The Al_2O_3 shell was applied by AIP hydrolysis, adapting an existing method.[55] Equal weights of Fe_3O_4@C-x and AIP (target 20 wt% γ-Al_2O_3) were added to roughly 120 times weight ethanol, sonicated, then heated to 45 °C for 12 h. Then, 100 times weight 5:1 EtOH:DI H_2O was added dropwise, stirred 1 h, heated to 78 °C and stirred 24 h. The solid was collected and washed 4 times in 50:50 DI H_2O:IPA, dried at 100 °C for 12 h, annealed in flowing N_2 at 600 °C (1 °C min^{-1}) for 5 h, then at 550 °C for 2 h in 5% H_2. This was repeated a second time.

Three batches of Fe_3O_4@C@γ-Al_2O_3 were made: FCA-1 and FCA-2 using Fe_3O_4@C-1, and FCA-3 using Fe_3O_4@C-2.

Reactor Schematics and Analytical Details

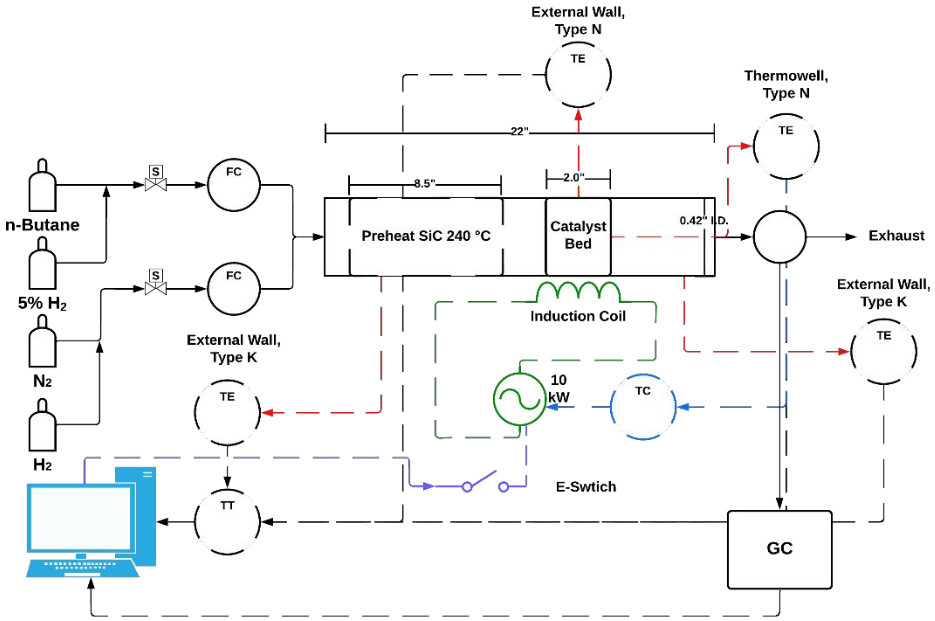

Figure S1. Dehydrogenation reactor schematic, RF-IH setup shown

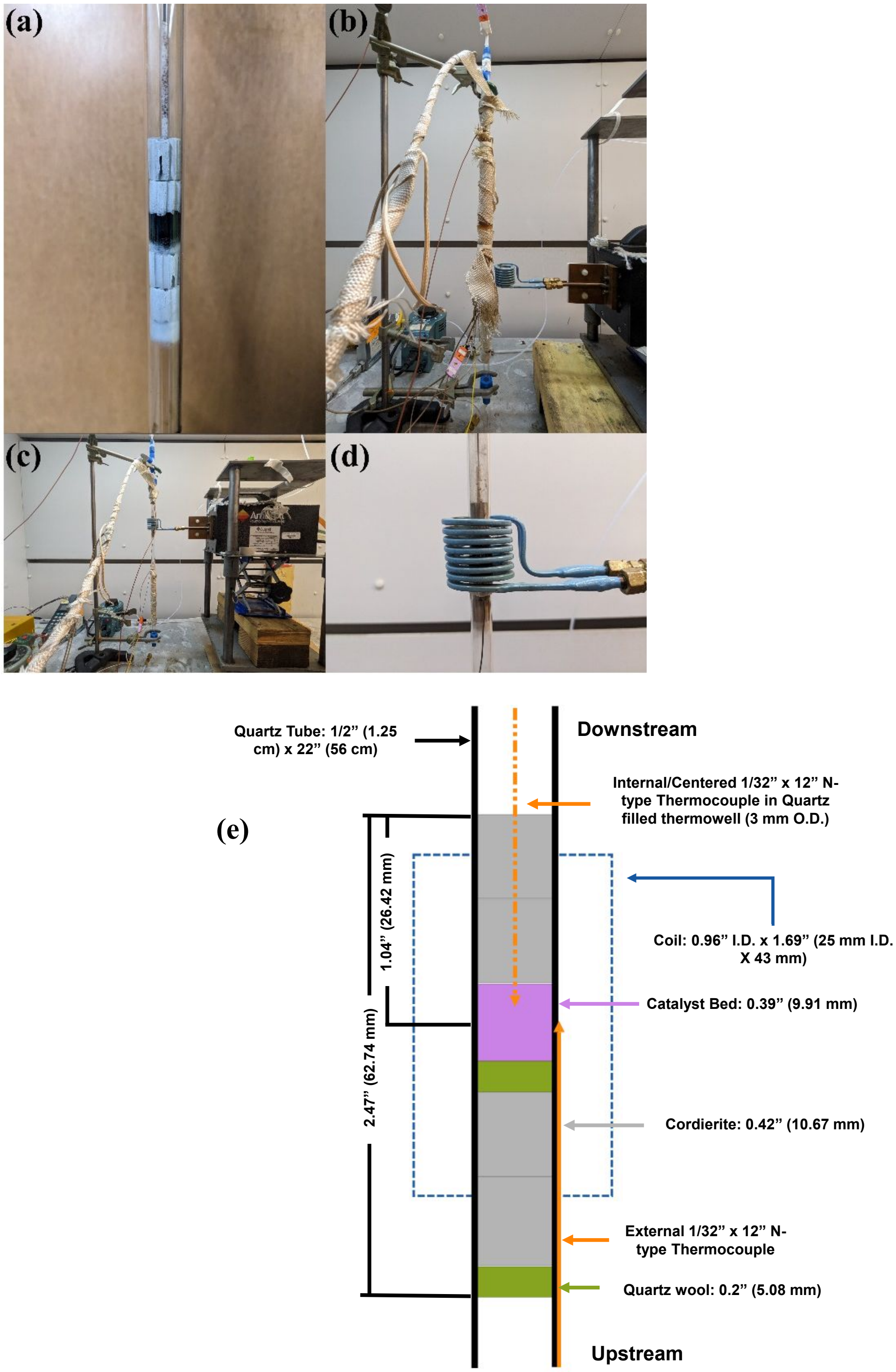

Figure S2. Pictures/schematics of the thermal and RF-IH reactor setup: (a) catalyst bed with internal thermocouple, (b) complete thermal reactor setup, (c) complete RF-IH reactor setup, (d) closeup of catalyst bed showing the coil and external thermocouple, (e) schematic of thermocouple placement.

Chromatographic Details for Pt and Pt-Sn Dehydrogenation Experiments

Equipment: HP 6890, Supel Q™-PLOT (30 m x 0.32 mm) column linked to a Chrompack PLOT Fused Silica CP-Al_2O_3/KCl (15 m x 0.32 mm) column

Carrier gas: hydrogen, 170.5 mL min^{-1} total flow

Injection loop volume: 50 uL

Front inlet: initial temp. 25 °C; pressure 12.9 psi; split ratio 40:1; split flow 160.0 mL min^{-1}

Front detector: FID at 150 °C; hydrogen flow 35.0 mL min^{-1}; makeup flow 10.5 mL min^{-1}; air flow 350.0 mL/min

GC oven program: initial temp. 28°C; initial time 20.0 min; ramp rate 2.0 °C min^{-1}; final temp. 80 °C; final time 8.00 min; total run time 54 min.

Figure S3. Chromatogram of a typical separation, dehydrogenation of n-butane over Pt or Pt-Sn catalyst

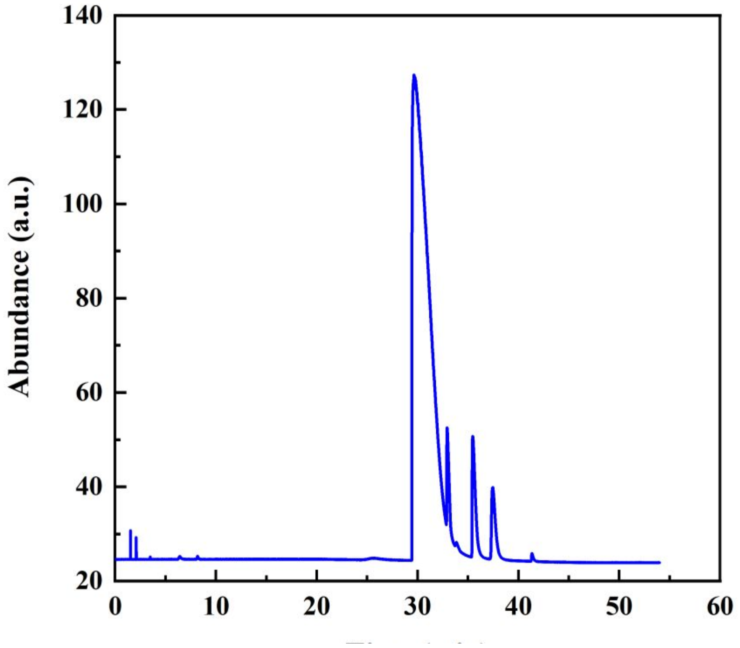

Chromatographic Details for VO_x Dehydrogenation Experiments

Equipment: Same as above

Carrier gas: hydrogen, 330.5 mL min^{-1} total flow

Injection loop volume: Same as above

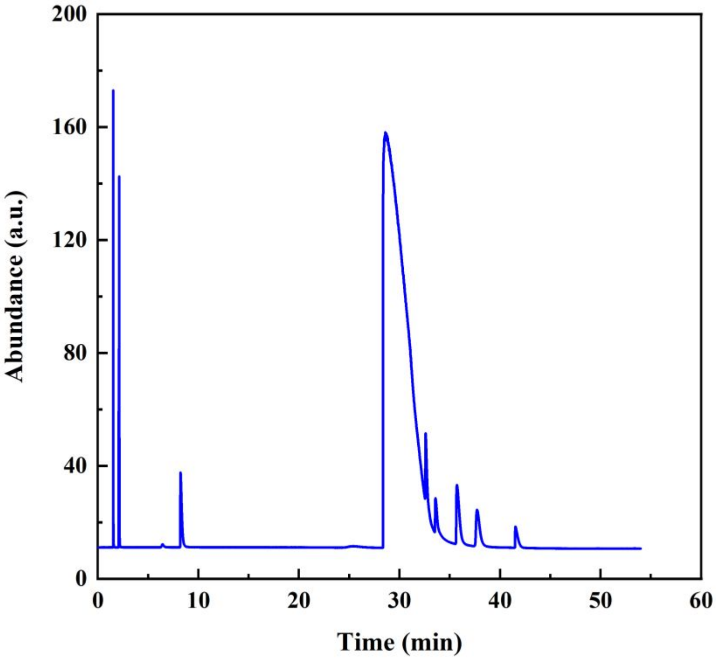

Figure S4. Chromatogram of a typical separation, dehydrogenation of n-butane over VO_x catalyst

Front inlet: initial temp. 25 °C; pressure 12.9 psi; split ratio 80:1; split flow 320.0 mL min^{-1}

Front detector: Same as above.

GC oven program: Same as above.

Supporting Analytical Work for Catalyst Syntheses

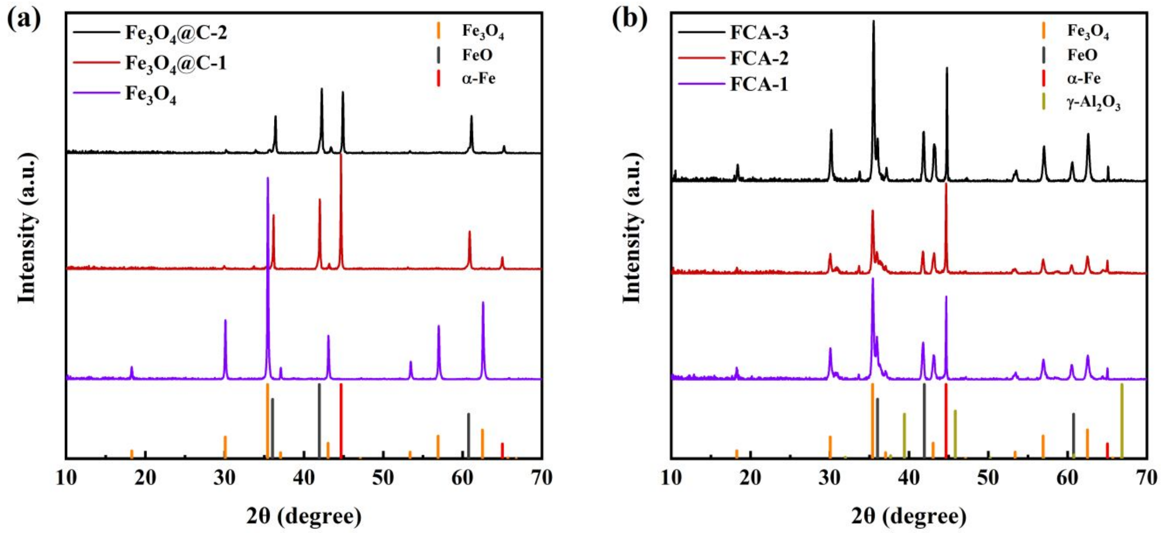

Figure S5. XRDs of: (a) initial Fe_3O_4 and Fe_3O_4@C core-shell supports; and (b) Fe_3O_4@C@γ-Al_2O_3

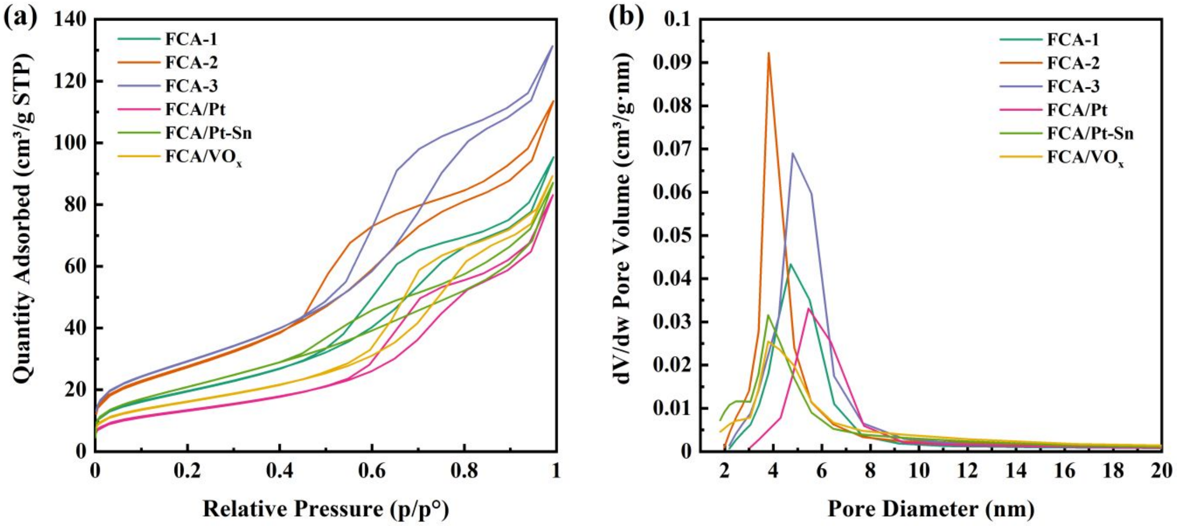

Figure S6. For the supports and the core-shell catalysts: (a) isotherm linear plots; and (b) pore diameter distributions

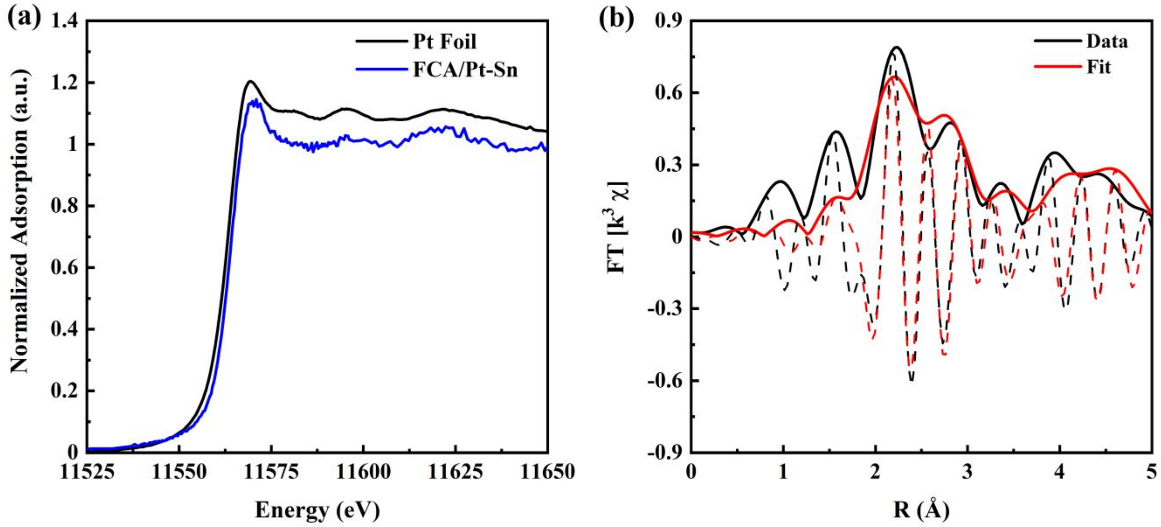

Figure S7. Pt L3 Edge (a) XANES and (b) XAFS of FCA/Pt-Sn

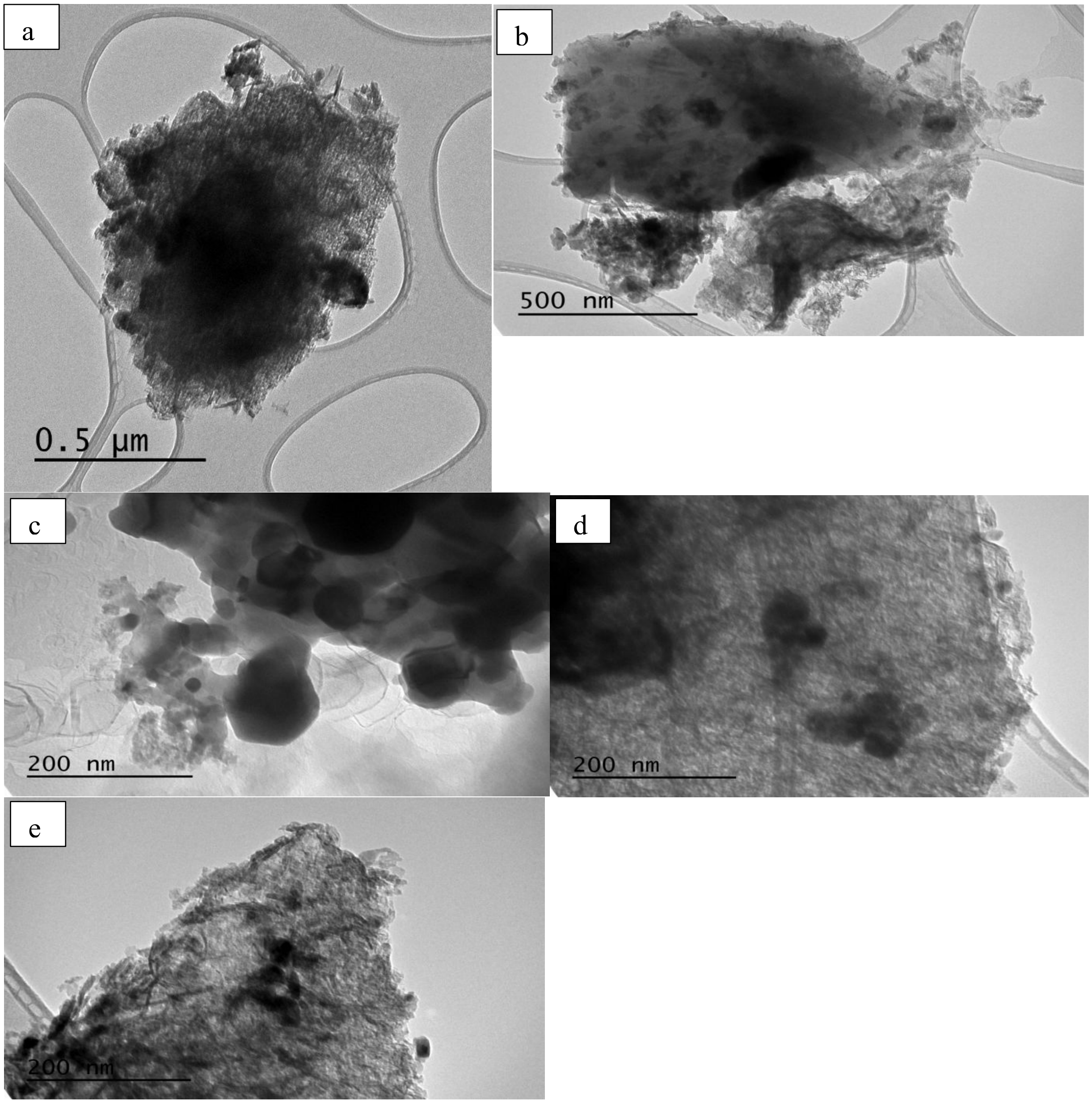

Figure S8. TEM images of core (Fe_3O_4)-shell (Al_2O_3) syntheses: (a) low magnification FCA-2; (b) low magnification FCA-3; (c, d, e) higher magnifications of FCA-3 with Fe_3O_4 average sizes (darker regions) of 63, 48, and 27 nm respectively.

Dehydrogenation Reactions Supporting Data

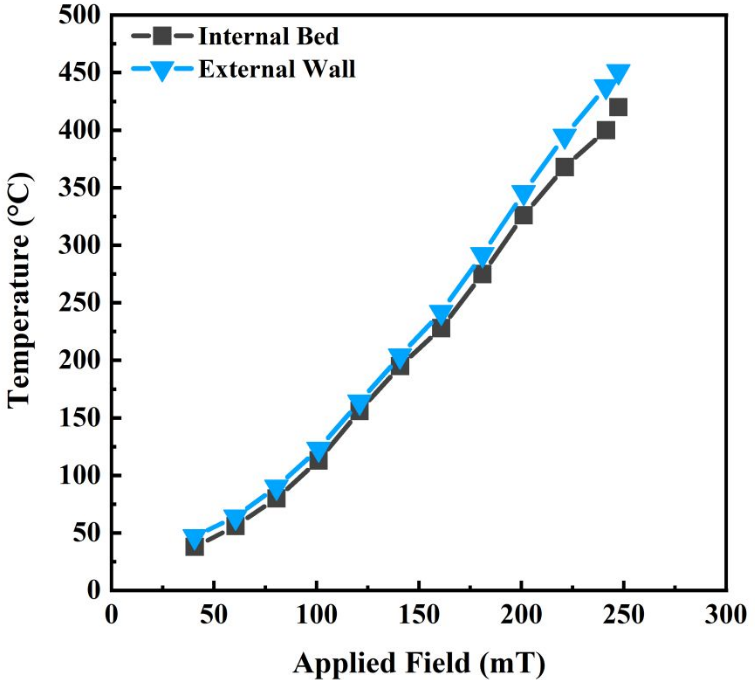

Figure S9. Internal bed and external wall thermocouple temperature calibrations up to the maximum possible applied field (247 mT)

The key question in the temperature measurements is how well the bed thermocouple reflects the induction heating of the catalyst adjacent to it rather than its own (possibly additional) induction heating and any EMF interference effects. An N-thermocouple is less magnetically susceptible than K-type.[1] The positive conductor for N-type is Ni-14.2% Cr-1.4% Si and the negative Ni-4.4% Si-0.1% Mg. Neither is ferromagnetic. For K-type the negative is Ni-Al, which is ferromagnetic.[1-2] The sheath for N-type is a Ni-Cr alloy, also not ferromagnetic, and is isolated from the thermocouple itself by a layer of MgO insulation.[2] The bed thermocouple assembly was in turn encased in a quartz thermowell surrounded by quartz sand.

As seen in Fig. S9, in the absence of catalyst the thermocouple also absorbs heat (75-80°C at 75 mT) in the absence of a catalyst bed. But the disparity between its heating and that of the catalyst bed is so great that it is reasonable to assume that when in contact with the bed its measured

value is much more reflective of the bed. Aside from the more thorough experiments cited in the paper (ref. 66 of the paper) with glass-sheathed thermocouples inside water, suggesting negligible EMF effects on the measurements, there are also the experiments of Smalcerz and Przylucki,[3] who employed (non-ferromagnetic) metal-insulated K-thermocouples, but without an additional glass sheath. They concluded based on experiments with the thermocouple positioned inside an RF-heated steel ingot that there was no significant error (not quantified by them, but definitely <5°C based on their raw data) due to the RF-IH even at high frequency (worked at both 50 Hz and 330 kHz) and up to >550°C, as long as: (a) there is a surrounding ferromagnetic medium; (b) the thermocouple is of the insulated variety. We emphasize that for our experiments: (a) there is a surrounding ferromagnetic (Fe in the catalysts) medium; (b) the thermocouple is of the insulated type; (c) there is also surrounding the thermocouple a quartz sheath and sand; (d) the thermocouple was N-type, which is less magnetically susceptible than the K-type used in both ref. 66 and by Smalcerz and Przylucki.

To further examine this point, we conducted individual measurements using both Fe_3O_4 and Fe nanoparticles (because, as shown, the Fe_3O_4 component of the catalyst is reduced during the dehydrogenation experiments) in the reactor, with a N_2 carrier gas, at various field strengths. The results are shown below, with two replicated experiments. In the top graph (Fig. S9) we used 0.75 g Fe_3O_4, and the entry "TC below" refers to the thermocouple below the level of the bed, in the quartz wool but still within the induction field. For a typical catalyst (e.g., FCA/Pt-Sn) the average initial field strength at nominal 555°C was 125 mT, the final 65 mT, while at nominal 505°C the initial was 75 mT, final 49 mT. From these results we conclude that even under these conditions (no H_2), the Fe_3O_4 is probably being reduced somewhat. But the main takeaway is the large difference in temperature between the two curves. In Fig. S10 in one set of experiments we

also placed the thermocouple still in contact with the Fe bed, but right at its bottom. In this case the measured temperatures are much higher and closer to the measurements at the axial bed center.

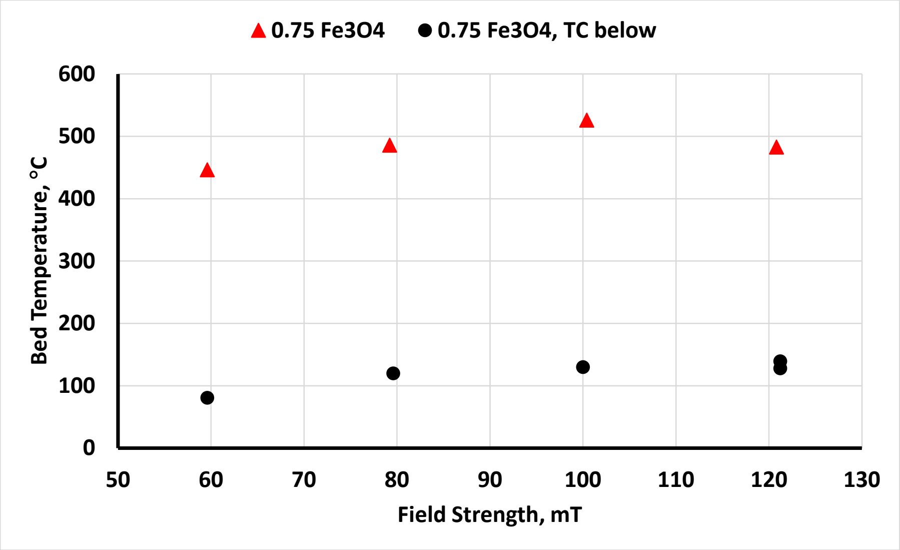

Figure S10: Thermocouple susceptibility measurements with 0.75 g Fe_3O_4. "TC below" means the thermocouple was placed below the bed, in the quartz wool.

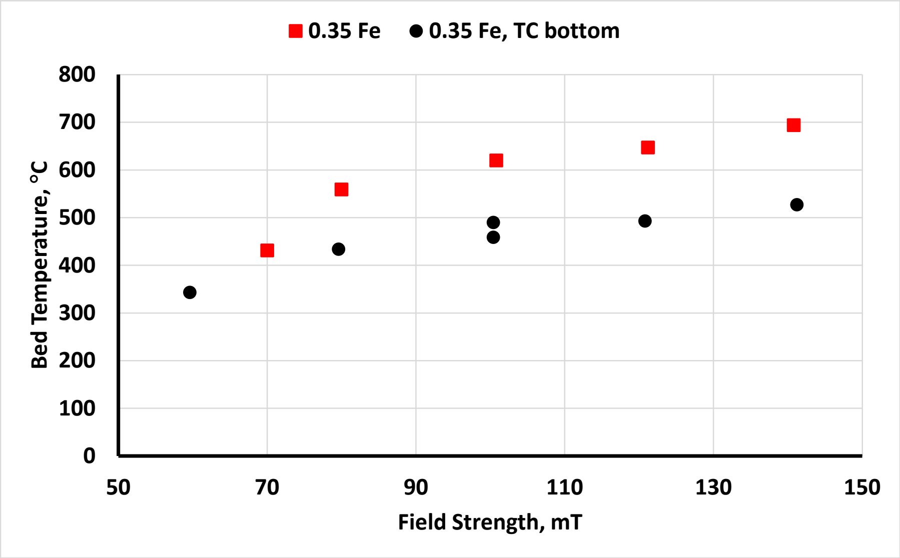

Figure S11: Thermocouple susceptbility measurements with 0.35 g Fe. "TC bottom" means the thermocouple was placed at the exact bottom of the bed, just above the quartz wool.

Note that we only used half the Fe in the second set of experiments. This was intentional, to simulate partial rather than complete Fe_3O_4 reduction. However, we also conducted experiments with the same amount of Fe as Fe_3O_4 (0.75 g), and in this case we found much higher temperatures.

We conducted further experiments using a series of salts with known melting points, and welding crayons, which melt at prescribed temperatures. The salts were mixed with the Fe_3O_4 or Fe in small quantity, while the welding crayon marks were placed on the outer reactor wall. For the final experiment of Fig. S10 (near 120 mT) we found that $Pb(NO_3)_2$ (melting point 470°C) melted while $PbCl_2$ (melting point 501°C) did not. This is exactly what would be expected if the measured thermocouple temperature was close to accurate. Two welding crayon marks (at 510 and 538°C) also did not melt.

For the experiments with Fe (Fig. S11), $PbCl_2$ melted at 101 mT, as did the 538°C pen mark, but not the 550°C pen mark. This is entirely expected assuming the thermocouple is correct. But $CuCl_2$ (melting point 598°C) did not fully melt. From Fig. S11 it is seen that the measured temperatures at this strength are right at the point where $CuCl_2$ could melt, but not significantly higher. Again, it appears that the temperatures are in the vicinity of the actual bed temperatures.

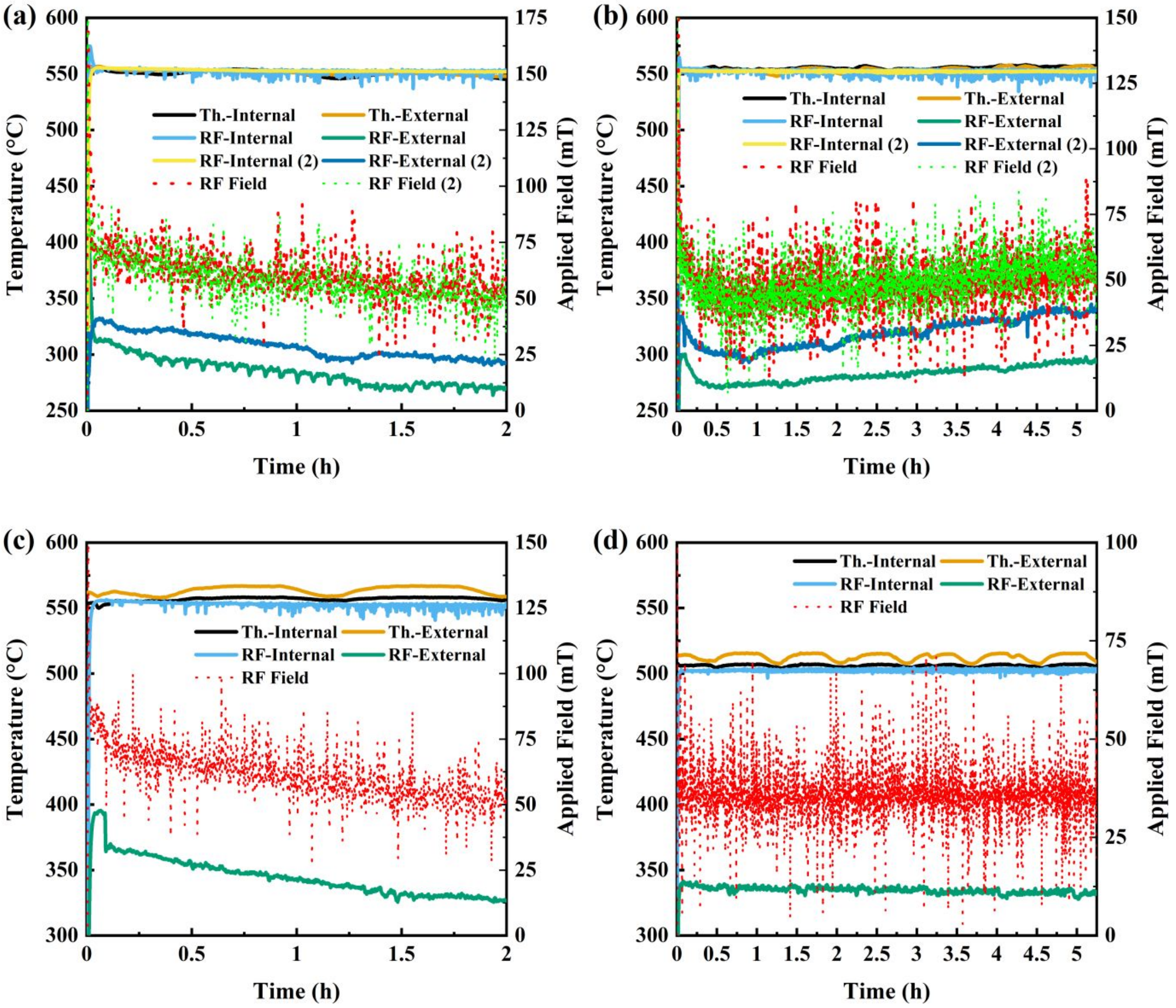

Figure S12. FCA/Pt internal bed and external wall temperatures, and applied field data for the thermal and RF-IH catalysts: (a) reduction of the 555 °C catalyst; (b) dehydrogenation at 555 °C; (c) reduction of the 505 °C catalyst; and (d) dehydrogenation at 505 °C

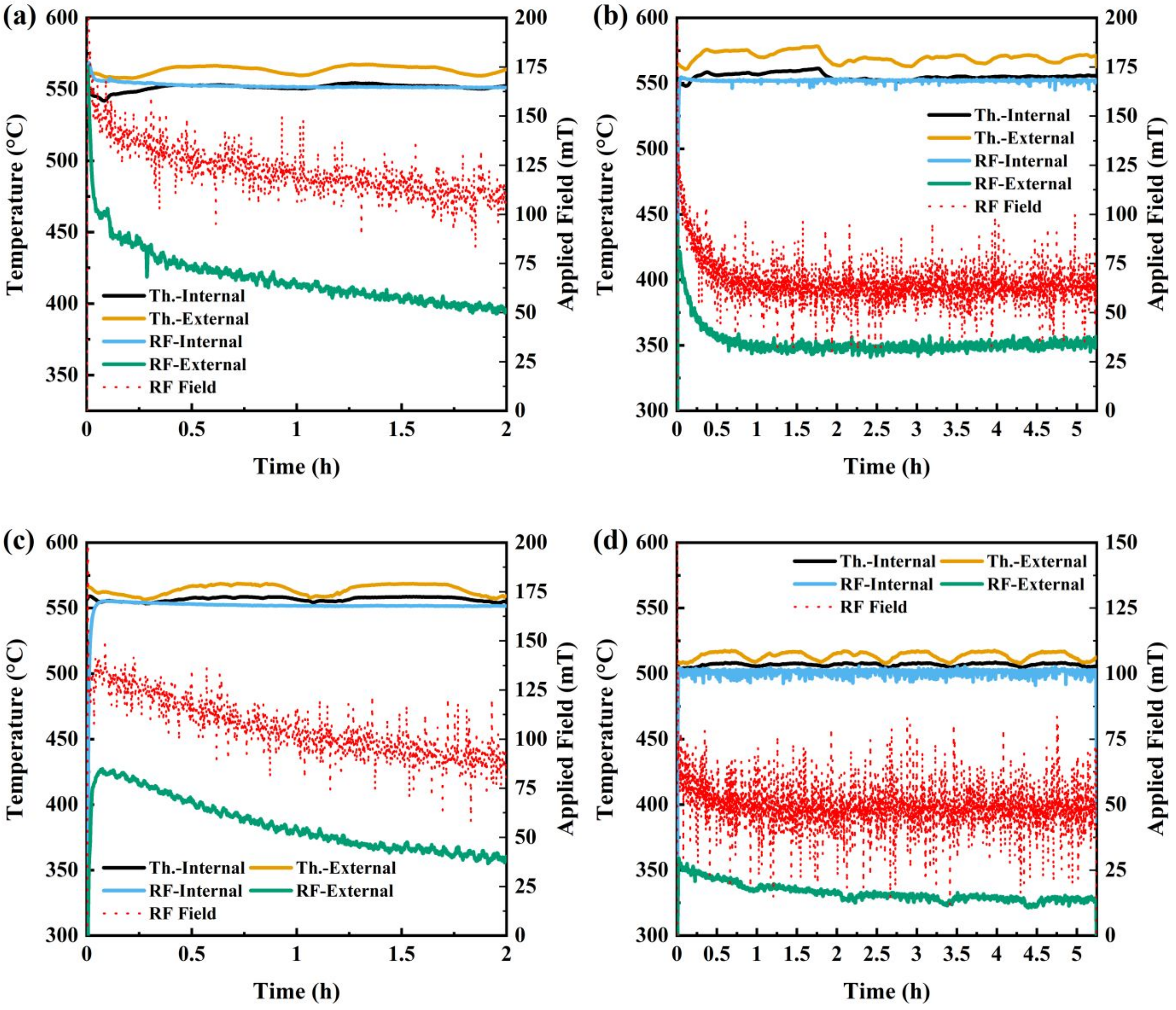

Figure S13. FCA/Pt-Sn Internal Bed, External Wall, and Applied Field data for the Thermal and RF-IH catalysts: (a) reduction of 555 °C catalyst; (b) dehydrogenation at 555 °C; (c) reduction of the 505 °C catalyst; and (d) dehydrogenation at 505 °C

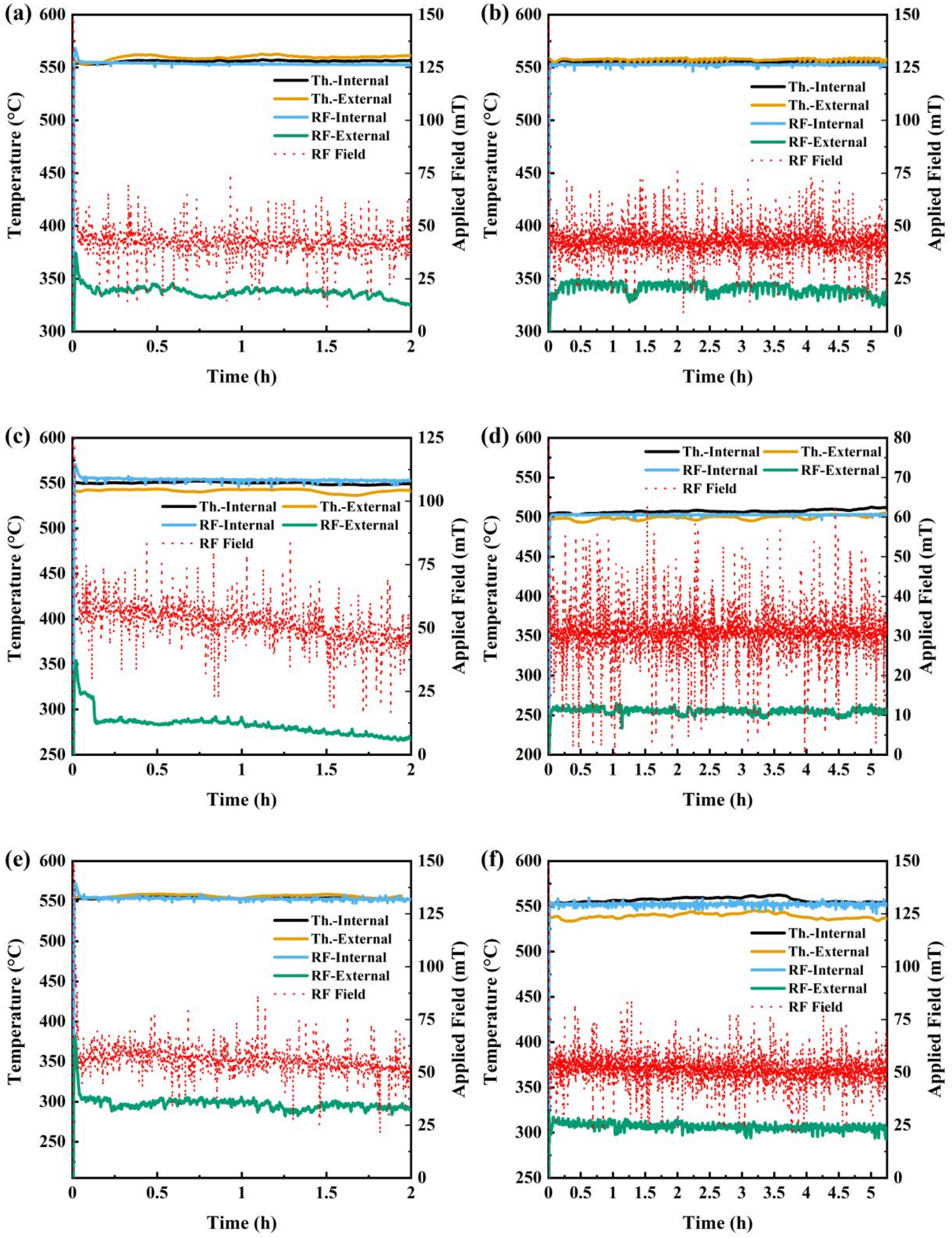

Figure S14. FCA/VO_x Internal Bed, External Wall, and Applied Field data for the Thermal and RF-IH catalysts: (a) reduction of 555 °C catalyst (2.04 h^{-1}); (b) dehydrogenation at 555 °C (2.04 h^{-1}) (c) reduction of 505 °C catalyst (2.04 h^{-1}); (d) dehydrogenation at 505 °C (2.04 h^{-1}); (e) reduction of 555 °C catalyst (1.22 h^{-1}); and (f) dehydrogenation at 555 °C (1.22 h^{-1})

EDS maps, porosimetry, SEMs and TEMs of used catalysts

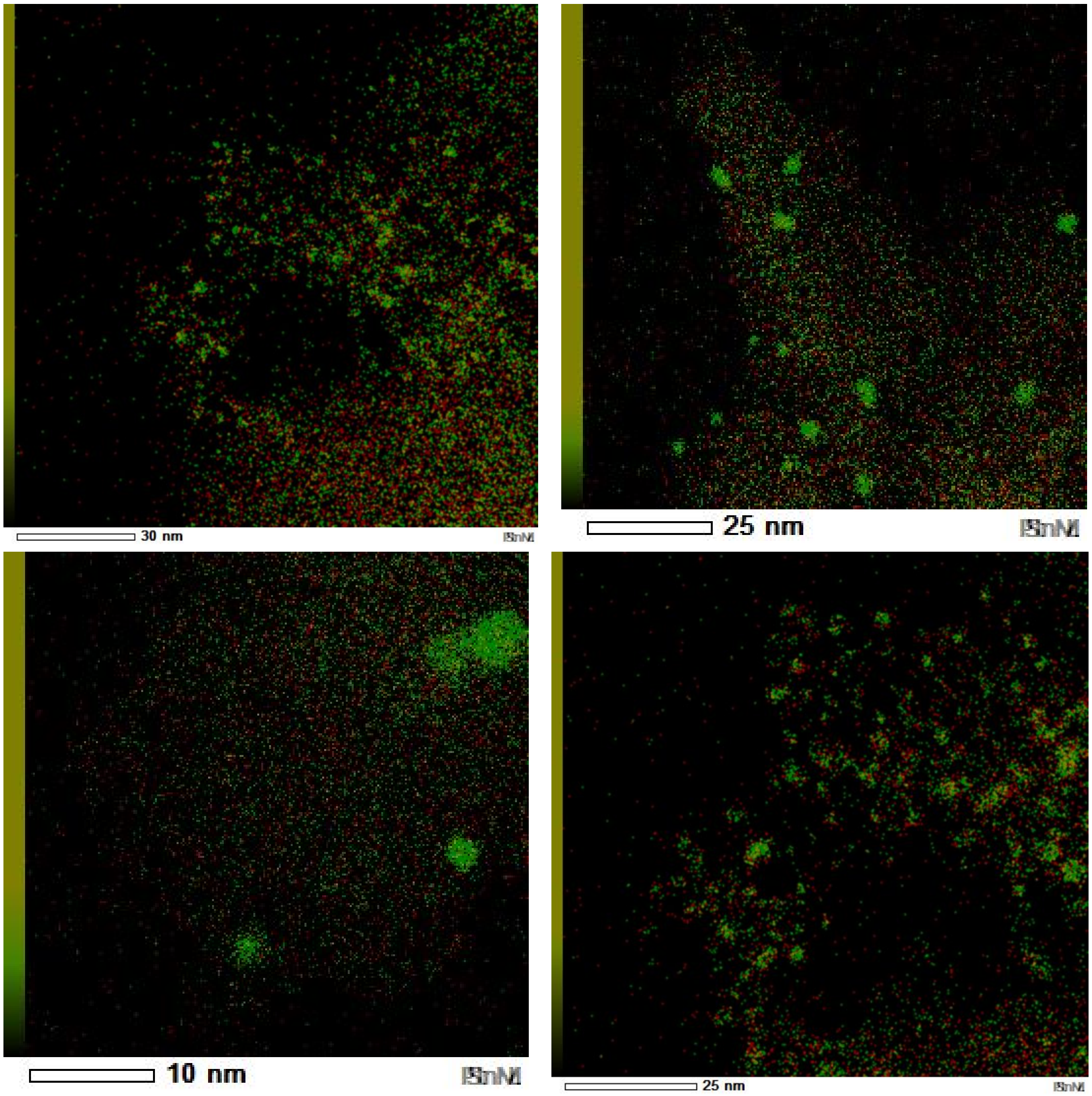

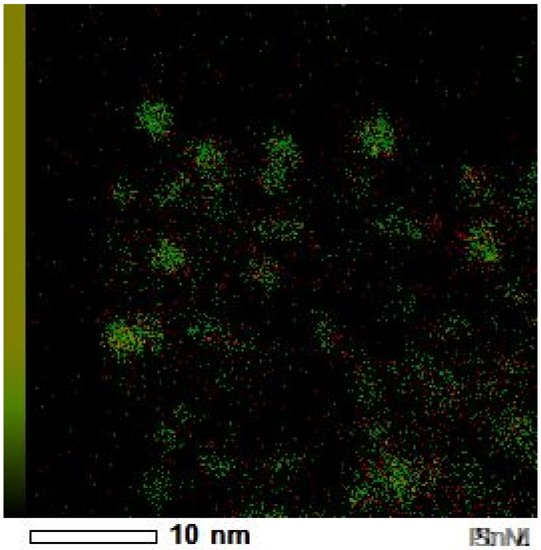

Figure S15. EDS maps of FCA/Pt-Sn, Sn (red) overlaid on Pt (green)

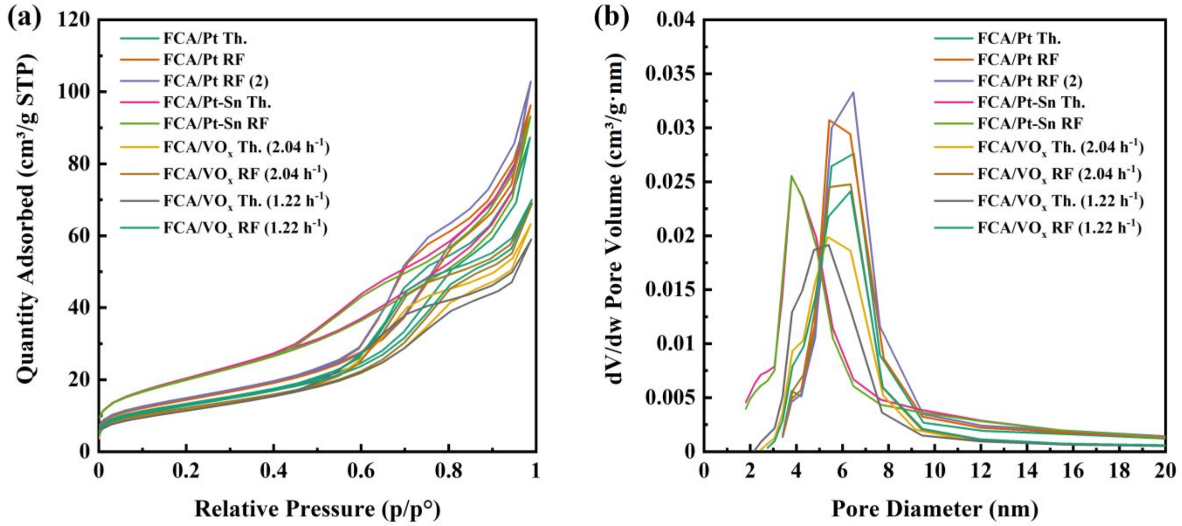

Figure S16. For the used catalysts: (a) isotherm linear plots; and (b) pore diameter distributions distributions

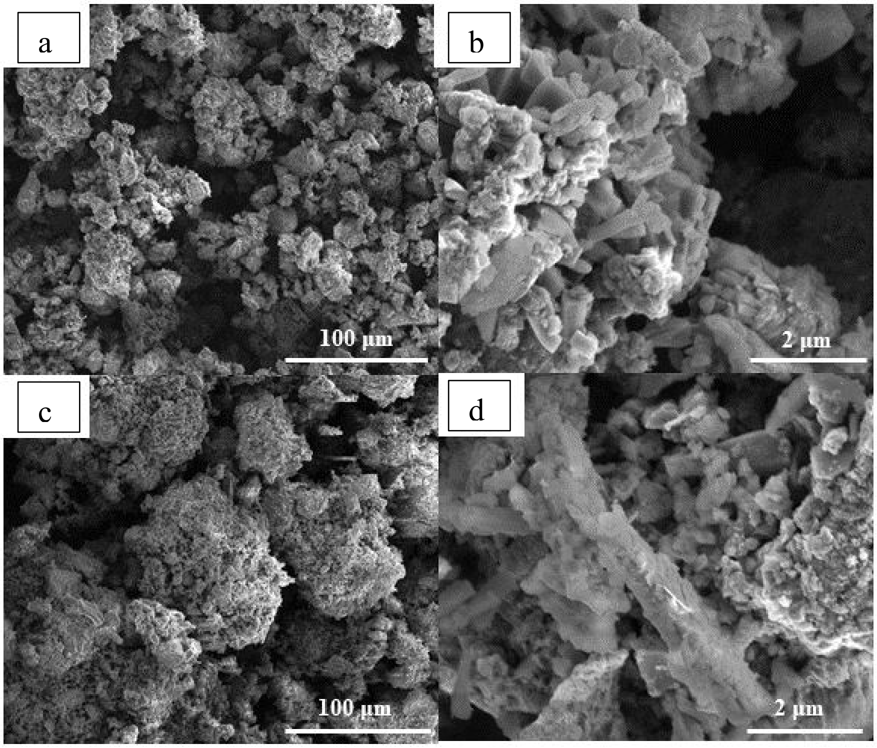

Figure S17. SEMs of used catalysts: (a) 550 °C FCA/Pt-Sn Th.; (b) same at higher magnification; (c) 550 °C FCA/Pt-Sn RF; and (d) same at higher magnification

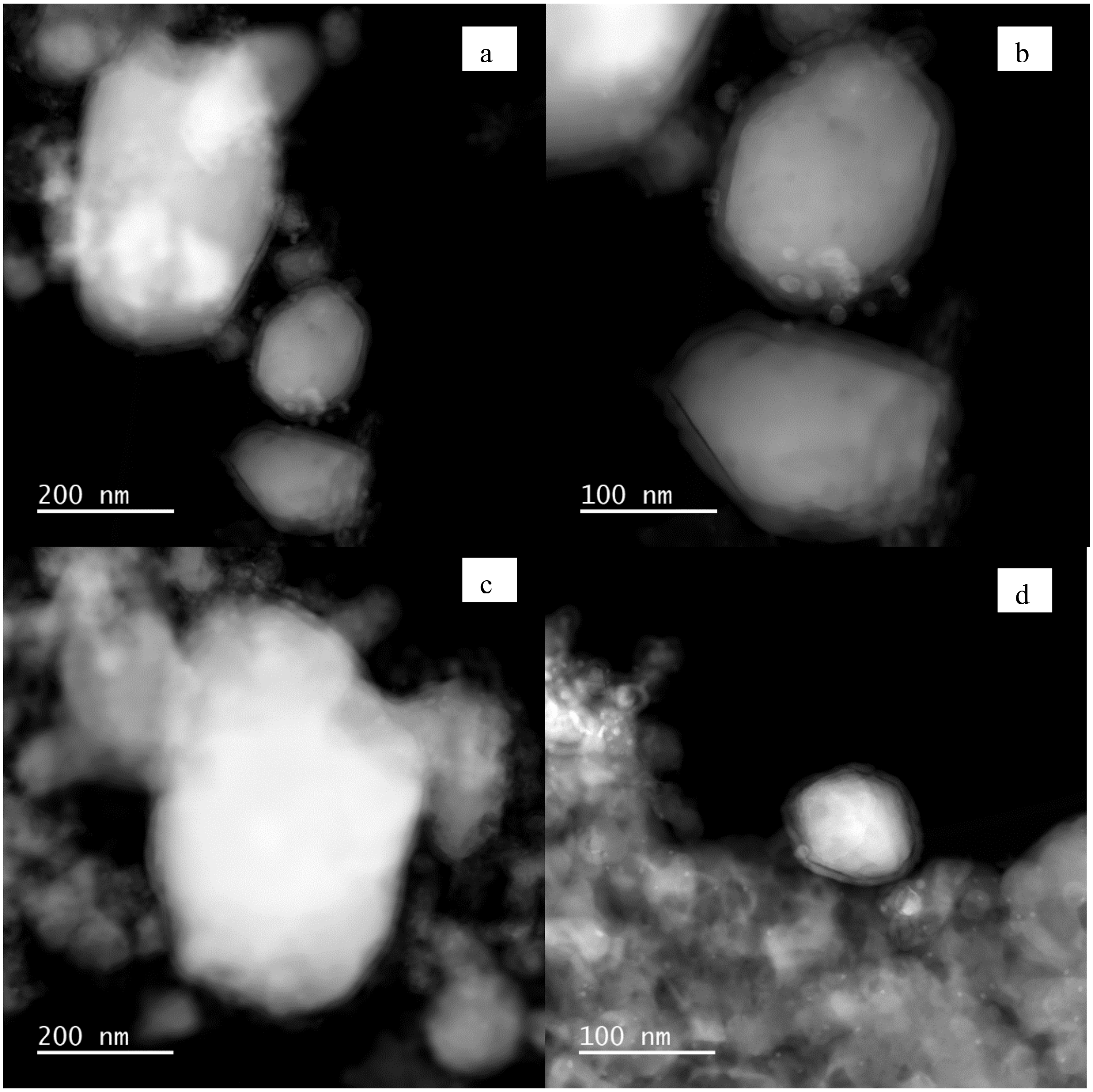

Figure S18. TEM images of used: (a, b) Pt/FCA-Th.; (c, d) Pt/FCA-RF

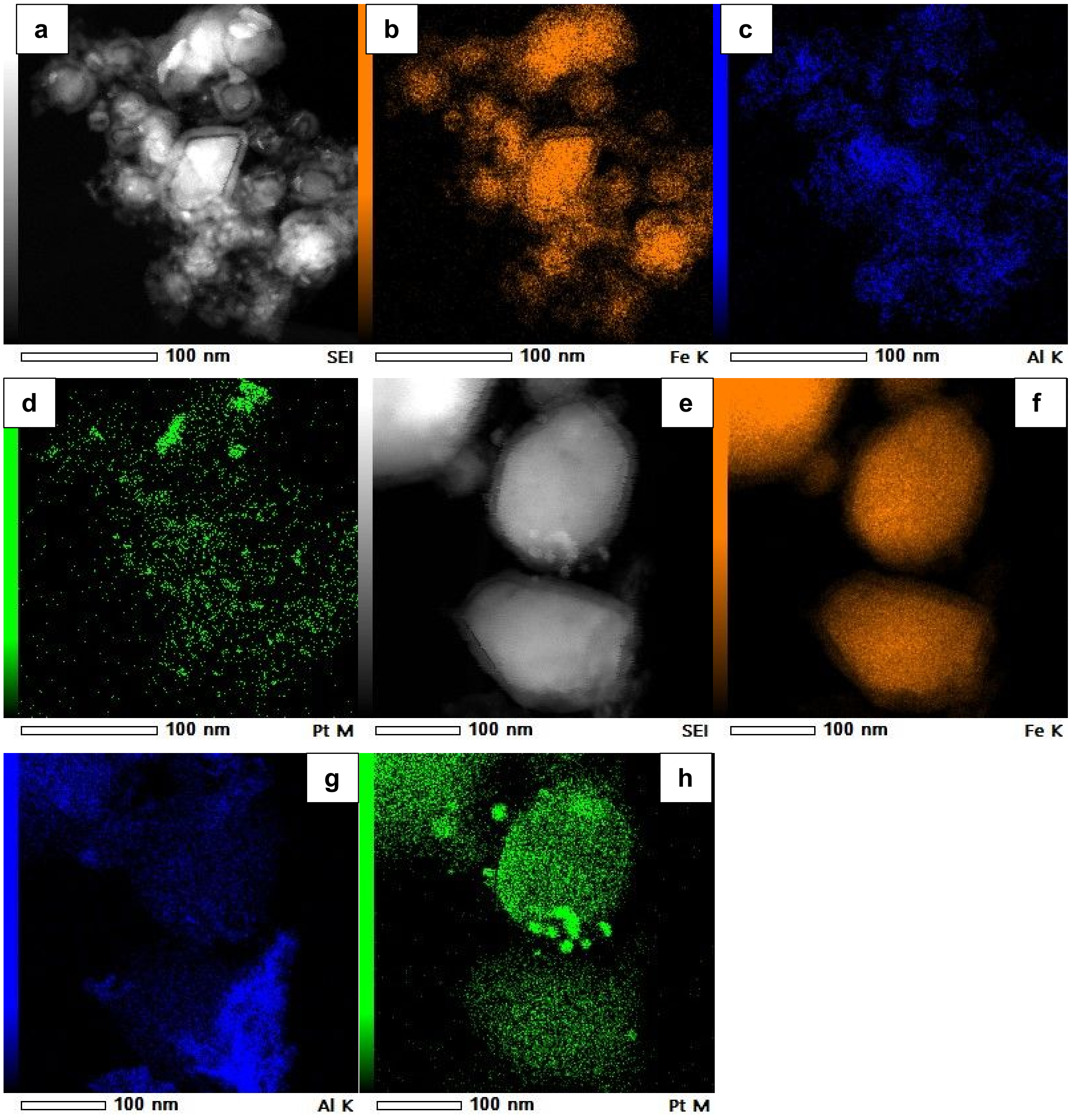

Figure S19. Scanned electron images and EDS maps of used FCA/Pt-RF (a-d) and used FCA/Pt-Th. (e-h) catalysts: (a, e) scanned electron images; (b, f) Fe EDS; (c, g) Al EDS; (d, h) Pt EDS

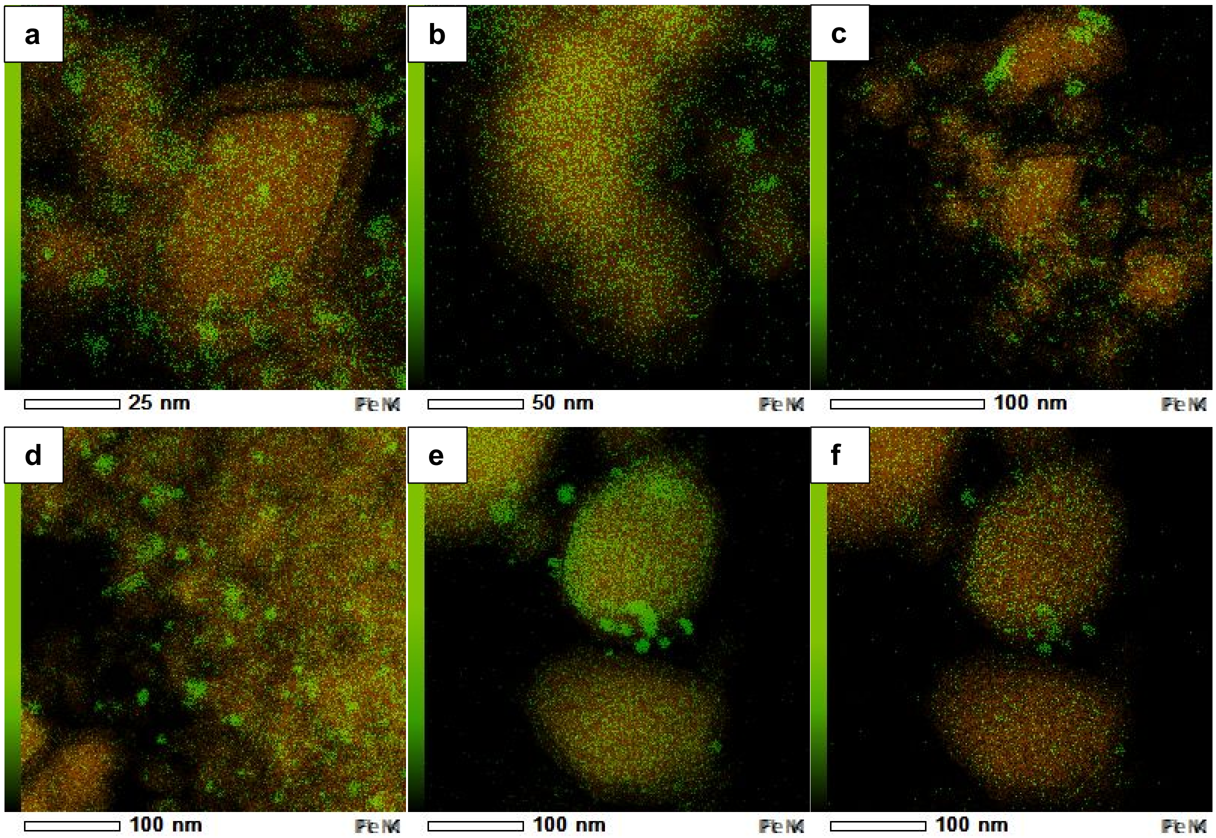

Figure S20. EDS maps of used FCA/Pt, Pt (green) overlaid on Fe (red): (a-c) FCA/Pt-RF; (d-f) FCA/Pt-Th

References

1. https://www.tc-inc.com/thermocouples/type-n-thermocouple.html (accessed 1/8/25)

2. https://assets.omega.com/spec/SUPER-OMEGACLAD-XL.pdf (accessed 1/8/25)

3. Smalcerz, A.; Przylucki, R. Impact of Electromagnetic Field upon Temperature Measurement of Induction Heated Charges. Int. J. Thermophys. 2013, 34, 667-679. DOI 10.1007/s10765-013-1423-1
